# Volume and surface methods for microparticle traction force microscopy: a computational and experimental comparison

**DOI:** 10.1039/d6sm00242k

**Published:** 2026-06-17

**Authors:** Simon Brauburger, Bastian K. Kraus, Tobias Walther, Cornelis Mense, Tobias Abele, Kerstin Göpfrich, Ulrich S. Schwarz

**Affiliations:** a Institute for Theoretical Physics, Heidelberg University 69120 Heidelberg Germany schwarz@thphys.uni-heidelberg.de; b BioQuant, Heidelberg University 69120 Heidelberg Germany; c Cavendish Laboratory, University of Cambridge CB3 0HE Cambridge UK; d Center for Molecular Biology of Heidelberg University (ZMBH), Heidelberg University 69120 Heidelberg Germany k.goepfrich@zmbh.uni-heidelberg.de; e Cluster of Excellence SynthImmune, Heidelberg University 69120 Heidelberg Germany

## Abstract

It is an essential element of mechanobiology to measure the forces of biological cells. In microparticle traction force microscopy, they are inferred from the deformation of elastic microparticles. Two complementary variants have been introduced before: the volume method, which reconstructs surface stresses from the displacements of fiducial markers embedded inside the particles, and the surface method, which infers stresses directly from the deformation of the particle surface. However, a systematic comparison of the two methods has been lacking. Here, we quantitatively compare both approaches using simulated traction fields representing biologically relevant loading scenarios. We find that the surface method consistently reconstructs traction profiles with substantially lower errors than the volume method, which suffers from displacement tracking and stress calculation at the surface. At high noise levels, however, the performance gap becomes smaller. To compare the performance of the two methods in a realistic experimental setting, we developed DNA-based hydrogel microparticles equipped with both fluorescent surface labels and embedded fluorescent nanoparticles, enabling the direct comparison of the two methods within the same system. Compression experiments produced traction profiles consistent with Hertzian contact mechanics and confirmed the trends observed in the simulations. We also show that despite large experimental deformations and strains (both up to 20 percent), linear elasticity theory should still be valid. While our computational workflow establishes a framework to apply both methods, our experimental workflow establishes DNA microparticles as versatile and biocompatible probes for measuring cellular forces.

## Abstract 2

Computer simulations show that the surface method performs better for microparticle traction force microscopy than the volume method. This is confirmed by experiments with DNA-based hydrogel microparticles.

## Graphical abstract

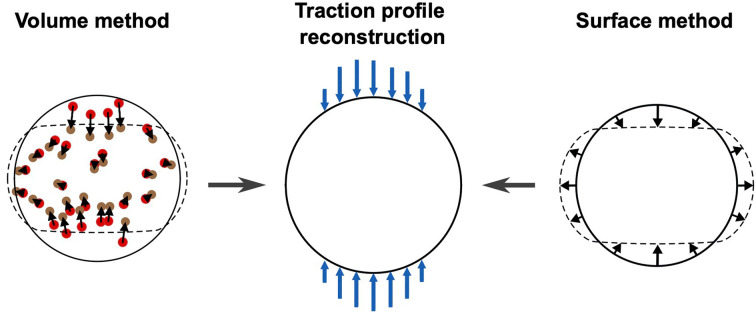

## 1 Introduction

The generation and sensing of physical force is an important element in many cellular processes, including cell differentiation, division and migration.^1^ During tissue morphogenesis, for example, cells are guided by the mechanical properties of their surroundings,^2,3^ while immune cells probe mechanical properties to select their targets.^4,5^ To measure the physical forces at work in such biological systems, a large range of different methods has been established in the field of mechanobiology.^6–8^

The standard method to measure forces of single cells is traction force microscopy (TFM).^9,10^ In the classical setup, a single cell is placed on a flat, elastic substrate with embedded fluorescent nanoparticles. The substrate is deformed by the cellular forces, causing measurable displacement of the nanoparticles. By tracking the nanoparticles close to the surface, the displacement field on top of the substrate is obtained, from which the traction field can be reconstructed using elasticity theory. The most efficient method for this purpose is Fourier transform traction cytometry (FTTC), which maximizes the agreement between predicted and measured data (inverse method) and inverts the relation between forces and displacement in Fourier space.^11^ The effect of the inevitable noise in the experimental data can be counteracted by regularization.^12^ Alternatively, one can directly map strain onto stress (direct method), but this requires taking image stacks and derivatives of noisy data.^13–15^ Today, TFM on flat substrates is a very mature method, which has been adapted to different cellular contexts. For both inverse and direct methods, software solutions are available in the public domain.^15–19^

TFM is also routinely applied to multicellular systems. A straightforward example is epithelial monolayers, for which TFM has been extended by monolayer stress microscopy, which predicts stresses in the monolayer from traction forces on soft elastic substrates.^20–22^ This approach can even be applied to intestinal organoids, if they are prepared to adhere to a flat substrate.^23^ To measure cell forces in full three-dimensional contexts, one usually embeds the system of interest in collagen gels.^24,25^ The mechanics of such gels, however, is less well defined, leading to lower resolution.

While all of these methods use spatially extended substrates to measure cell traction, one can also employ small deformable probes embedded into the system of interest. This approach has been pioneered for fluorescently labeled oil droplets.^26^ A huge advantage of this approach is the possibility of these force sensors to be used inside multicellular systems such as embryos, tumor spheroids or organoids.^27,28^ One disadvantage of the oil droplet system is that as a fluid, it can only measure normal and not shear forces.

Over the last years, there has been a growing effort to replace fluid droplets with spherical gels, called microparticles, typically made from polyacrylamide, the standard material used for spatially extended substrates in TFM.^29–32^ This approach is called microparticle traction force microscopy (MP-TFM)^29^ and comes with its own challenges, mainly due to its small system size compared with spatially extended systems. Yet, it is a very appealing approach, because it combines the advantages of traditional TFM and the fluid droplet method: MP-TFM enables the detection of shear forces *in situ*, which means inside living biological systems.

**Fig. 1:**
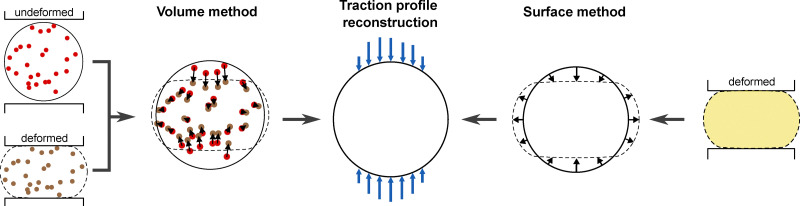
Volume and surface methods in microparticle traction force microscopy (MP-TFM). In the volume method (left), the three-dimensional displacement field is obtained by tracking fluorescent nanoparticles inside the microparticle from the undeformed reference state (red) to the deformed state (brown). Surface tractions are calculated using the direct method, which directly maps strain to stress. In the surface method (right), the geometry of a deformed microparticle (usually homogeneously fluorescent, yellow) is compared to an assumed spherical reference. The traction field is then inferred from the surface displacement using an expansion in vector spherical harmonics. In a simple variant of the surface method, only radial displacements are being considered. Both volume and surface methods ultimately infer a surface traction profile (center), but use different information and assumptions.

Two main strategies have emerged to reconstruct traction fields from elastic microparticles, as depicted in Fig. 1. The first approach, introduced by Mohagheghian *et al.*,^30^ follows the classical paradigm of traction force microscopy. Fluorescence images of marker nanoparticles embedded throughout the microparticle are recorded both in an undeformed reference state and in a deformed state. From these images, the three-dimensional displacement field *u⃑*(*r⃑*) inside the particle volume is obtained using particle tracking or volume correlation algorithms. The traction field is then calculated from the displacement field by evaluating spatial derivatives of *u⃑* and applying the constitutive relations of linear elasticity. This is known as the direct method in conventional TFM.^15^ In the following, we refer to this variant of MP-TFM as the volume method.

The second approach, which we will refer to as the surface method, is closer to the fluid droplet method, relies solely on the deformation of the particle surface, and has been pioneered by Vorselen *et al.*^29,33–35^ From this boundary geometry, surface displacements are inferred relative to an assumed spherical reference configuration. These surface displacements are expanded in vector spherical harmonics, which enable the conversion into a traction field.^31,32,36^ To achieve higher accuracy, the displacement field can then be further refined by iteratively minimizing an appropriate energy functional of the traction and displacement fields, albeit at higher computational cost.^29^ One disadvantage of this method is that the reference configuration is not measured, but assumed. In particular, in the fast variant without refinement, only radial displacements are considered.^31,32^

The two methods differ fundamentally in the type of displacement information they use (volume- *versus* surface-levels) and in the assumptions they make, especially about the reference configuration. Therefore, it is not evident *a priori* which strategy should provide more accurate or robust traction estimates under realistic experimental conditions. A systematic comparison of both methods has not been conducted before. Here, we present a systematic comparison between the two methods and introduce an experimental system (DNA-based hydrogel microparticles with appropriate fluorescent markers) that allows us to test these two methods also in practice. Although in principle other pipelines might be developed for MP-TFM, *e.g.*, an inverse variant of the volume method, here we restrict our treatment to the two commonly used and already optimized pipelines.

This work is structured as follows. First, we computationally compare the accuracy of the two different methods in recovering a known traction profile on the surface of a sphere. For both methods, we simulate suitable experimental data for three different traction profiles mimicking relevant biological scenarios and calculate the errors in recovering the prescribed traction profile. Overall, we find that the surface method achieves lower reconstruction errors than the volume method, making better use of the information about the relevant deformations near the microparticle surface. We attribute this to surface effects of the correlation algorithm used to track the fiducial markers in the volume method, leading to inaccuracies that result in an underestimation of tractions in the area where the force was actually applied. In order to experimentally validate our findings, we then introduce a new experimental system for MP-TFM, namely DNA-based hydrogel microparticles (DNA-HMPs). DNA-HMPs have recently been shown to exhibit elastic solid-like material properties and can be produced in a controllable manner with stiffnesses tunable between 30 Pa and 6 kPa. DNA-HMPs are further easily equipped with guest molecules for chemical stimulation of surrounding cells and stably embedded into organoid and spheroid systems, enabling directed tissue engineering.^37,38^ Employing DNA-HMPs thus offers a biocompatible and tunable approach for 3D force measurements *in vitro*. We test the DNA-HMPs on both the volume and the surface method by compressing them inside a custom-made microwell setup, where the DNA-HMPs are deformed using a weighted glass slide to recreate a Hertzian contact scenario. Both methods retrieve the overall shape of the traction profile. The surface method achieves a closer match to the corresponding analytical solution, in agreement with the simulation results. Finally, we demonstrate through computer simulations with hyperelasticity, that even for the large deformations and strains used in our experiments, linear elasticity is still justified.

## 2 Materials and methods

### 2.1 Volume method

Displacements were estimated using the fast iterative digital volume correlation (FIDVC), developed by Franck *et al.*,^39,40^ and available on GitHub.^41^ FIDVC is based on the digital volume correlation (DVC) algorithm to align two images *I*(*x*, *y*, *z*) (reference) and *Î*(*x*, *y*, *z*) (deformed), containing gray level intensity values. We first divide the image *I* into subvolumes *V*_*i*_ of integer voxel size lengths *l*_*x*_, *l*_*y*_, *l*_*z*_, also referred to as window size. For each given subvolume *V*_*i*_, we now try to estimate a single displacement vector *u⃑* = (*u*_*x*_, *u*_*y*_, *u*_*z*_) by evaluating a performance metric for different trial displacements *u⃑*_*t*_ and choosing the one with the best result. As performance metric, we use a cross-correlation function that characterizes the degree of similarity between two pictures by multiplying the intensity values of all voxels in the volume. In our application, these two pictures consist of the reference picture *I* and the deformed picture *Î*, where to the latter *u⃑*_*t*_ has been applied reversely. We sum over all the voxels of the subvolume *V*_*i*_:1
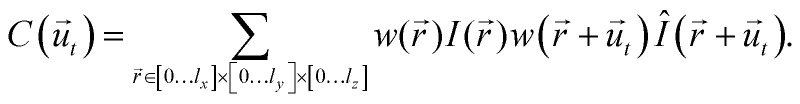


A weight function *w*(*r⃑*) is introduced to compensate for the frequency response of the moving average filter. Furthermore, to increase computational speed and obtain more accurate results, we evaluate eqn (1) in Fourier space and use a third order polynomial Gaussian peak algorithm.^39^

To decrease computation time, improve resolution, and enable the detection of larger displacements, the procedure described above has been extended into a multi-step iterative algorithm called fast iterative DVC (FIDVC).^40^ The main idea is that we do not start with the final resolution, but with a large window size and thus a lower resolution in order to find a coarse grained displacement field. This displacement field is then reversely applied to the images, the window size is halved, and the procedure is repeated until the desired resolution or accuracy is reached.

Once the displacement field has been obtained from the measurement data using the FIDVC, the next step is to calculate the traction from the displacement field. For the volume method, we use the direct method, corresponding to directly mapping displacements to strains and then strains to stresses. The strain tensor describes relative deformations. Assuming small relative deformations, its linear approximation, which significantly simplifies the following calculations, is given *via*2
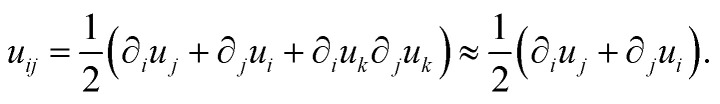


Assuming that our material is an isotropic Hookean solid, we can define the stress tensor that connects the strain and material properties. It is characterized by the Lamé parameters *μ* and *λ*:

3 *σ*_*ij*_ = 2*μu*_*ij*_ + *λδ*_*ij*_*u*_*kk*_.

Evaluating the stress tensor across the surface of the body by multiplying it with the normal vector *n⃑* of the surface at that point yields the traction *T⃑*, which describes the stresses acting on the surface of the body:

4 *T*_*i*_ = *σ*_*ij*_*n*_*j*_.

Thus, in order to retrieve the traction, we first need to compute the spatial derivatives of all displacement components to obtain the strain tensor. Each displacement field component as returned from the FIDVC is given as a 3D array with one value for each voxel, so the data is discretized with voxel spacing *d*. This allows us to express the differentiation as a matrix operator, or (finite difference) kernel, performed for each point by the summation of element-wise multiplication of the operator with the surrounding data points. In this work we use the 5-tap kernel:^42^5
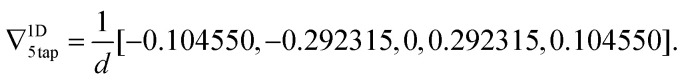


For higher dimensions, the kernel needs to be reshaped for each direction to represent the respective derivative. It is also common to additionally perform averaging over the dimensions orthogonal to the direction of the derivative, resulting in, what is known as, the optimal-5 kernel.^39^

Applying the kernels described above gives us the strain tensor *u*_*ij*_ for each voxel in Cartesian coordinates. In the next step, we calculate the stresses *σ*_*ij*_ using eqn (2) and (3) at each voxel. In order to retrieve tractions, we need to evaluate the stresses with the corresponding normal vector at the surface. We use a Gauss–Legendre quadrature (GLQ) mesh as set of points to perform the evaluation (see Fig. S1) because the GLQ mesh is also used extensively in the surface method.^51^ Due to the surface effects, we evaluate the traction not on the surface of the sphere, but on a smaller sphere with the evaluation radius *r*_e_ < *r*_0_ (more details in Section 3.2). We interpolate *σ*_*ij*_ from the surrounding voxels onto the point of the evaluation sphere surface using the RegularGridInterpolator function of numpy. Finally, tractions are obtained with eqn (4), using the corresponding normal vector of each point on the grid. The traction components, which now represent the magnitude in terms of Cartesian unit vectors, are also transformed to spherical coordinates.

### 2.2 Surface method

Force reconstruction by the surface method is based on minimizing an energy functional which was proposed by Vorselen *et al.* in 2020,^29^ and uses the decomposition into spherical harmonics introduced by Wang *et al.* in 2019.^36^ Note that this approach is only possible for finite systems like MPs, so that the surface carries a lot of information. The main idea has some similarity with the inverse method of traditional TFM, because it also minimizes a functional. For MP-TFM, the functional consists of three terms representing various considerations. First, any residual traction on a region which is known to be traction-free is penalized. Second, the elastic energy should be minimal to account for the minimum energy principle. Third, we want to remove aliasing effects by penalizing high-frequency components. As a linear combination, we obtain:6
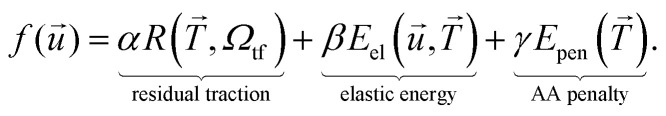


The parameters *α*, *β*, and *γ* are defined in Table S1; these choices were shown to provide optimal results in the original implementation of the surface method.^29^ To evaluate this functional quickly, it is necessary to use a fast way to convert from *u⃑* to *T⃑*, as the terms in the functional have an explicit dependence on *T⃑*. The solution is to choose a basis for the displacement function space where each basis vector *u⃑*^(*K*)^ corresponds to a basis vector *T⃑*^(*K*)^. To construct this basis, we start by looking at the space of possible solutions. These need to obey the equilibrium condition

7 ∇·*σ* = 0,

as we assume that the sphere is in mechanical equilibrium. In the next step, we use a harmonic potential ansatz for the potential:^43^8
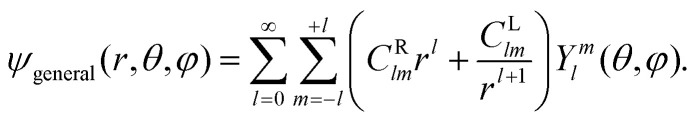


We disregard the terms ∼1/*r*^*l*+1^, as they diverge at the origin (*r* = 0). The corresponding displacement solutions are then given by the Papkovich–Neuber solutions:^43^9
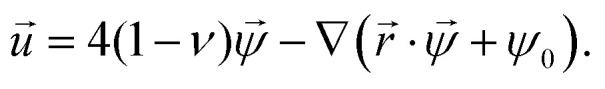


These provide a basis for the relevant displacement solutions, whose angular part is represented as a superposition of spherical harmonics. Evaluating eqn (2)–(4), we find a similar decomposition for the traction *T⃑*. As the decomposition for each basis vector *u⃑*^(*K*)^ and *T⃑*^(*K*)^ is fixed, the conversion between them reduces to a simple matrix multiplication (for detailed derivation see Note S1^36,52^). This decomposition enables us to adopt a vector representation of the full displacement field (see Note S2^53,54^). In this way, we can rewrite the functional and its derivatives in terms of matrices, facilitating a minimization of the functional in a reasonable amount of time. This minimization happens in the subset of displacement functions that match the surface profile (obtained by either experiment or simulation) and is performed using the conjugate-gradient method (detailed explanation in Note S3^55,56^).

### 2.3 Data simulation

To obtain a reference solution which can be used to simulate experimental data, we use the analytical solutions of axisymmetric stress problems presented by Mietke *et al.*^44^ This represents a special case of the general solution presented in Section 2.2. A given axisymmetric traction profile *T⃑*(*θ*) on the surface of a sphere can be decomposed *via* a set of coefficients *a*_*n*_ and *e*_*n*_, using the orthogonal Legendre polynomials *P*_*n*_:10
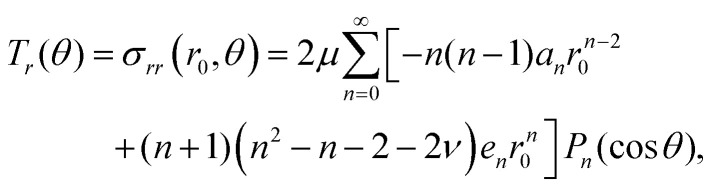
11
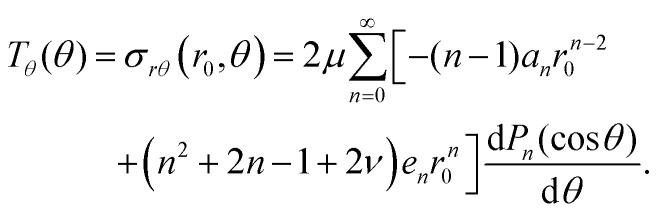


These coefficients directly determine the full solution for the displacement of the sphere resulting from the traction:12
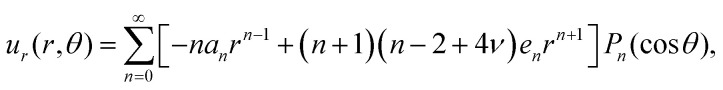
13
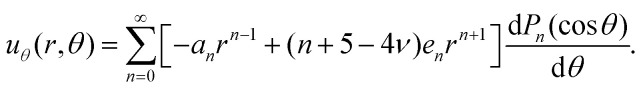


For any boundary condition *T⃑*(*θ*), we can now quickly calculate an analytical solution for the full deformation field *u⃑*(*r*, *θ*, *φ*) inside the sphere. The described decomposition is of infinite size, so we need to set a cutoff order for the sums *n*_max_ = 50 was found to be a sufficient compromise between computational speed and accuracy (see Fig. S2).

To simulate image data for the volume method, we create two 3D arrays; *I*(*x*, *y*, *z*) for the undeformed and *Î*(*x*, *y*, *z*) for the deformed image. Its entries represent intensity values simulating the fluorescence measurements. To obtain these values, the algorithm creates uniformly randomly distributed nanoparticle positions inside a sphere of radius *r*_0_. Each voxel's intensity value is then generated by evaluating a squared Gaussian point spread function (PSF) for every nearby nanoparticle. For the deformed picture, we apply the deformation field obtained in eqn (12) and (13) to each of the nanoparticles and repeat the remaining procedure. In both cases, we add Gaussian displacement noise with amplitude *σ*_disp_ = 0.17 px to the nanoparticle positions before the evaluation of the PSF, resembling experimentally observed noise.^45^ The full procedure is explained in more detail in Fig. S3. If not stated otherwise, we use the simulation parameters as given in Table S1. Resolution as well as microparticle and nanoparticle properties closely resemble the experimental conditions in the original volume method publication.^30^

For the surface method, we generate *N* = 2000 surface points using a Fibonacci spiral lattice, which distributes successive points by the golden angle 
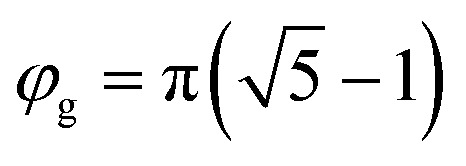
. This yields a nearly uniform areal density across the entire sphere with a mean surface spacing of 
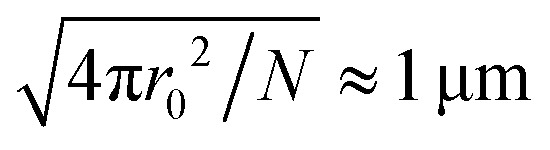
, matching the expected resolution of the surface reconstructions (a smoothing function with window size 1 µm was used in the original surface method implementation^29^). In contrast to the GLQ grid (used for the actual minimization and efficient traction evaluation by the spherical harmonics approach), the Fibonacci lattice avoids the polar oversampling inherent in latitude–longitude grids, and reconstruction performance between polar and equatorial force application is expected to be unbiased. For each point, we apply the calculated displacement field to obtain the positions of the deformed surface. Noise is implemented as surface roughness, added as a spherical random field in a spectral approach. Random spherical harmonic coefficients *a*_*lm*_ are drawn from a Gaussian distribution with zero mean and a continuous power spectrum

14 *C*_*l*_ ∝ (*l* + 1)^*β*^,

with power-law exponent *β* and minimum degree *l*_min_ = 2 (suppressing monopole and dipole terms to prevent global radius shifts and net displacement offsets). The coefficients are re-normalized so that the root-mean-square (rms) radial perturbation of the resulting field equals a prescribed relative amplitude *σ*_*r*_ (expressed as a fraction of the reference radius *r*_0_). The perturbed surface is obtained by scaling each point's radial coordinate by (1 + *δ* (*θ*, *φ*)), where *δ* is the roughness field interpolated at the Fibonacci point locations *via* bicubic interpolation on the GLQ grid. To characterize the traction-free region, we provide a boolean array. Its entries, each representative for one point, indicate if it is in the traction-free area (true) or not (false). This array is used to calculate the matrix **P** for the functional (see Note S2). Visualizations for an exemplary Fibonacci grid with applied traction field, traction-free regions, and roughness field are given in Fig. S4 and S5. For the evaluation of the functional, the spherical harmonic cutoff was chosen as *l*_max_ = 20 to maintain feasible runtimes: changing *l*_max_ from 20 to 30 increased the runtime per period from 5–10 seconds to 90–150 seconds, without a substantial gain in accuracy. In this case, there is effectively no need to include the anti-aliasing term in the functional, which only applies for SH components with *l* > 20; for the sake of completeness we still included it in the surface method section. For very localized profiles, it is necessary to increase *l*_max_, depending on the area of force application, see discussion. We quantify the dependence of the reconstruction quality on *l*_max_ in Fig. S6, confirming *l*_max_ = 20 as a reasonable choice. If not stated otherwise, the simulation parameters as given in Table S1 are used. The same material parameters as for the volume method were chosen to ensure comparability.

### 2.4 Error calculation

To quantify the accuracy of the recovered traction profiles, we assess the normalized mean absolute error (NMAE). In our analysis pipeline, the traction data is evaluated on a GLQ grid with points *x*_*i*_(*θ*_*i*_, *φ*_*i*_) for both surface and volume method. Choosing *θ*_*i*_ ∈ [0, 180°], the NMAE for a traction component is then given by15
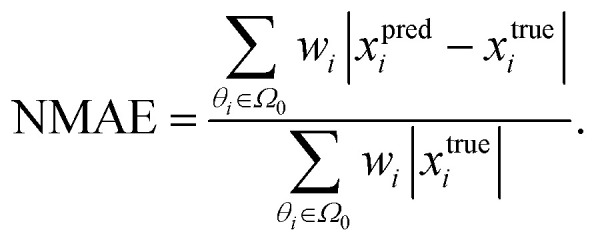


The weights *w*_*i*_ = sin *θ*_*i*_ correspond to the GLQ weights, accounting for the significantly higher point density near the poles due to the smaller spherical surface element d*A* = sin *θ*d*θ*d*φ*.

This *L*_1_-based error metric was chosen for its increased robustness against local noise-induced fluctuations compared to *L*_2_-based metrics. By performing a global normalization with the mean absolute true traction, rather than pointwise normalization, the metric remains well-defined and avoids artificial amplification of errors in regions where the ground-truth traction approaches zero.

### 2.5 Fabrication of DNA-based hydrogel microparticles

DNA was purchased either from Integrated DNA Technologies (unmodified DNA, purification: standard desalting) or Biomers (modified DNA, purification: HPLC). All DNA strands used to form the DNA-HMPs, apart from fluorophore-labeled strands, were diluted in 10 mM Tris (pH = 8) and 1 mM EDTA (Sigma Life Science) to yield 800 µm stock solutions. Fluorophore-labeled strands were diluted in MilliQ water to yield 800 µm stock solutions.

All DNA sequences are listed in Table S2. The DNA stock solutions were stored at −20 °C. Annealing of the DNA nanostars (A/B) was achieved by mixing the three respective DNA single-strands A-1, A-2, A-3 or B-1, B-2, B-3 at equimolar ratios resulting in 150 µm solutions of the DNA nanostars A and B. In all experiments, 4 mol% of cyanine-3 (Cy3)-labeled strands (B-1-Cy3) were added for visualization by confocal fluorescence microscopy. Phosphate-buffered saline (PBS; Gibco) solution at a final concentration of 1× as well as a final concentration of 10 mM of MgCl_2_ were added for annealing. The nanostars A and B were annealed in a thermal cycler (BioRad) by heating the samples to 85 °C for 3 min and subsequently cooling to 20 °C at an increment rate of 0.1 °C s^−1^. To create DNA-HMPs, the nanostars A and B were then mixed at 30 µm final concentration together with the DNA linker strand at 90 µm concentration in a solution of 1× PBS and 10 mM MgCl_2_. Fluorescent nanoparticles (fluorescein-tagged fluoresbrite yg microspheres (size: 0.3 µm), Cat. No. 24051, Polysciences) were added at 1 vol% to the above solution to create trackable markers (nanoparticles) inside the DNA-HMPs.

The DNA-HMPs were then formed in a droplet-templated manner following the encapsulation of the above solution into water-in-oil droplets with an oil-shell consisting of 2 wt% perfluoropolyether–polyethylene glycol (PFPE–PEG, RAN Biotechnologies) dissolved in HFE-7500 (Iolitex Ionic Liquids Technologies). Encapsulation was achieved by adding the aqueous solution on top of the oil phase in a volumetric ratio of 1 : 3 in a microtube (Eppendorf, typically 50 µL aqueous to 150 µL oil phase) and vortexing.^37,38^ The droplets were then stored at 21 °C room temperature for 72 h to allow the DNA-HMPs inside the droplets to fully assemble. After that, the DNA-HMPs were released from the water-in-oil emulsion by first adding the buffer solution of 1× PBS and 10 mM of MgCl_2_ at 1.5× of the initial volume on top of the droplet emulsion, and subsequently breaking the emulsion. To break the droplet emulsion, 1*H*,1*H*,2*H*,2*H*-perfluoro-1-octanol (Merck) was added on top of the buffer and droplet emulsion, and the mixture incubated for 30 min. The released DNA-HMPs in solution were then transferred to a new microtube for further use.

To equip the DNA-HMPs with a lipid coating, small unilamellar vesicles (SUVs) were formed. They were produced by mixing lipids (99% 1,2-dioleoyl-*sn-glycero*-3-phosphocholine (DOPC, Avanti Polar Lipids, Inc.) and 1% Atto-633-labeled 1,2-dioleoyl-*sn-glycero*-3-phosphoethanolamine (Atto-633-DOPE, Atto-Tec GmbH)) dissolved in chloroform (Honeywell) in a glass vial. The solvent was then removed under nitrogen gas and the glass vial placed into a desiccator for 20 min. The lipids were resuspended at a concentration of 10 mM in 1× PBS (Gibco) by shaking the glass vial at 900 rpm at room temperature for 10 min. From this solution, SUVs were extruded through a polycarbonate filter (100 nm pore size, Whatman) with 21 passages. The extruded SUVs were stored at 4 °C. All lipids were stored at −20 °C in chloroform.

Separately, cholesterol-tagged A-nanostars (A-chol) were prepared by annealing the single strands A-1-chol, A-2 and A-3 at equimolar ratios resulting in a 150 µm solution of A-chol. Prior to mixing, A-1-chol strands were incubated at 60 °C for 1 min to break up any aggregates of the cholesterol. Briefly, 50 µL of a DNA-HMP suspension was centrifuged using a C1008-GE myFUGE mini centrifuge (Benchmark Scientific) for 1 min, removing 30 µL of supernatant after centrifugation. The DNA-HMP pellet was then mixed with a final concentration of 5 µm A-chol and incubated for one hour to ensure uptake of A-chol into the DNA-HMPs. The DNA-HMPs were then washed three times with a 1× PBS and 10 mM MgCl_2_ solution before adding SUVs at a final concentration of 1 mM lipids on top of the DNA-HMPs, incubating at 4 °C overnight. Following three more washing steps after the overnight incubation using 1× PBS and 10 mM MgCl_2_ buffer, the lipid-coated DNA-HMPs were ready to use.

### 2.6 Fabrication of the microwell trapping device

Microwells for DNA microparticle trapping were fabricated using a custom-engineered setup, consisting of a Polygon digital micromirror device (DMD) pattern illuminator (MIGHTEX Polygon 1000) coupled to an inverted fluorescence microscope (Axio Observer 7, Carl Zeiss AG). The system was controlled by custom-written scripts based on Python 3.12.4 and the ZEISS ZEN Macro Environment. Printing was performed with a 5× objective (Plan-Apochromat 5×/0.16 M27, Carl Zeiss AG), defining the field of view for each exposure. Binary illumination masks consisting of square microwell frames and a three-letter alphabetical barcode were generated programmatically. To pattern the full sample area, adjacent 5× tiles were sequentially exposed by looping over the entire probe area, with the barcode letters automatically incremented for each microwell and tile. Illumination was provided by a built-in 385 nm LED, and each tile was exposed for 800 ms. Samples mounted on the microscope consisted of a 3D-printed PLA-based four-well chamber slide glued to a silanized 24 mm × 60 mm × 170 µm glass slide using two-component adhesive (eco-sil, picodent). The glass slides were silanized to allow for covalent binding of the printed microwells. For this, glass slides were cleaned by sonication in ethanol for 15 min, plasma-activated for 7 min at 200 W using ambient air (PVA TePla 100, PVA TePla AG), and subsequently immersed in a solution of 2% v/v trimethoxysilylpropyl methacrylate (TMSPMA) in toluene (300 mL) overnight, prior to washing in ethanol and MQ water. The wells were filled with a photoresist comprising 50 v% PEGDA 575 (Sigma Aldrich) in MQ water, 5 g L^−1^ lithium phenyl-2,4,6-trimethylbenzoylphosphinate (LAP, Sigma Aldrich), 5 g L^−1^ ascorbic acid (Sigma Aldrich), and 10 g L^−1^ tartrazine (Sigma Aldrich).

### 2.7 DNA-HMP deformation and imaging

The printed microwells were coated for 5 min with poly(vinyl-alcohol) (PVA, 50 mg mL^−1^, Sigma Aldrich). The wells were subsequently washed 3× by rinsing with a buffer solution of 1× PBS and 10 mM MgCl_2_. To soak the wells in the same buffer solution, 200 µL of the buffer remained in the wells after washing. Prior to imaging, 10 µL of the DNA-HMP suspension was evenly added onto the buffer-soaked wells and allowed to settle for 20 min. Next, a likewise PVA-coated glass coverslide (diameter: 10 mm, Carl Roth) was added onto the wells. The DNA-HMPs in the wells were imaged on an LSM 900 Zeiss confocal fluorescence microscope (Carl Zeiss AG). Using a Plan-Apochromat 20×/0.8 air M27 objective, a tile-scan of the entire area containing the microwells was taken and later used as a reference for DNA-HMP deformation. Suitable DNA-HMPs were then selected and the corresponding positions were saved. Using a 63×/1.2 W korr objective with oil immersion, reference *z*-stacks of the DNA-HMPs in the microwells were acquired using resolution-optimized Airyscan mode and 3× digital zoom at 512 px *xy*-resolution with 200 nm *z*-steps. The factor to convert voxels (pixels) to microns is approximately 0.099 for the *x*- and *y*-directions and 0.2 for the *z*-direction, resulting in an anisotropic resolution that is worse in *z* by a factor of about 2. To deform the DNA-HMPs, a 3D-printed circular weight (polylactic acid, white, diameter = 9 mm, 0.5 g) was placed on top of the glass coverslide, pushing it down and onto the DNA-HMPs. Next, *z*-stacks of the deformed DNA-HMPs were collected in the same manner as the reference stacks. Lifting off the adapter, the DNA-HMPs were allowed to relax from their deformed state to an undeformed one over several minutes and imaged again using the same settings, allowing us to observe the DNA-HMPs during relaxation. Mechanical characterizations of the DNA-HMPs were also performed to measure relevant elastic parameters (see Fig. S7).

### 2.8 Preprocessing of DNA-HMP images

To obtain suitable image pairs from experimental data for the correlation algorithm in the volume method workflow, the undeformed and deformed images of the nanoparticle configurations inside the DNA-HMPs were aligned with each other, including centering, cropping, and thresholding steps using custom Python scripts. To recover a set of points describing the microparticle surface for the initial radial displacement field guess for the surface method, the fluorescent information of the Cy3-tagged DNA network was analyzed, including blurring and thresholding in ImageJ,^46^ and surface reconstruction steps in GeoV.^47^ The detailed procedures are explained in Fig. S8–S10.

### 2.9 Finite element simulations of hyperelasticity

In order to check the validity of linear elasticity in the reconstruction procedures, simulations with the finite element method (FEM) were performed using the software package FEniCSx, which allows for easy use through the unified form language for weak formulations.^48,49^ Traction-indentation curves were simulated using both linear elasticity theory (LET) and nonlinear elasticity theory (NLET), the latter implemented using the neo-Hookean model. The microparticle is meshed in PyGmsh as a sphere with a radius of 10 µm, with Young's modulus 1.5 kPa, and Poisson's ratio 0.45. Surface displacement fields (*cf.*eqn (12) and (13)) are entered as a Dirichlet boundary condition for displacement of the mesh nodes, after which the remaining displacement field is solved subject to the constitutive relation. A similarly discretized version of eqn (3) is used for the LET-model.

To solve for the internal displacement field in the case of hyperelasticity, the first Piola–Kirchhoff (PK1) stress tensor for the NLET model is derived from strain energy density16
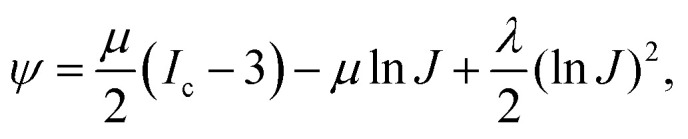
17
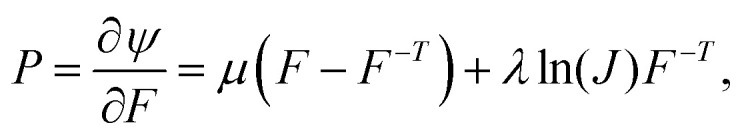
using the deformation gradient tensor *F*, volume ratio *J*, and first invariant *I*_c_.^50^ The resulting nonlinear system is solved using a Newton scheme. Its residual is defined in weak form with displacement field *u* as trial function and a test function *v*, as18
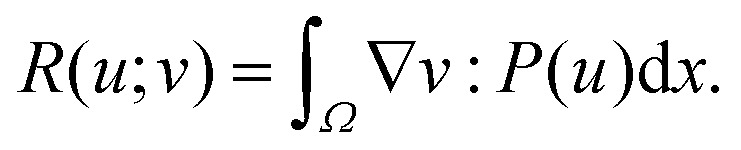


After convergence, the total magnitude of PK1 traction is computed using the direct method following19
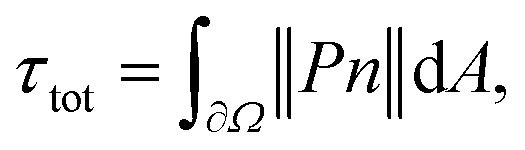
wherein *n* denotes the surface normal of the reference configuration. The expression for stress from eqn (3) was used to compute traction magnitude for LET. Linescans were performed for each type of deformation (Hertzian contact, indenter, ring), comparing the total integrated Piola traction between the models. Visualizations of the simulated deformations and traction profiles were made in ParaView.

## 3 Results and discussion

### 3.1 Performance comparison using simulated traction fields

**Fig. 2:**
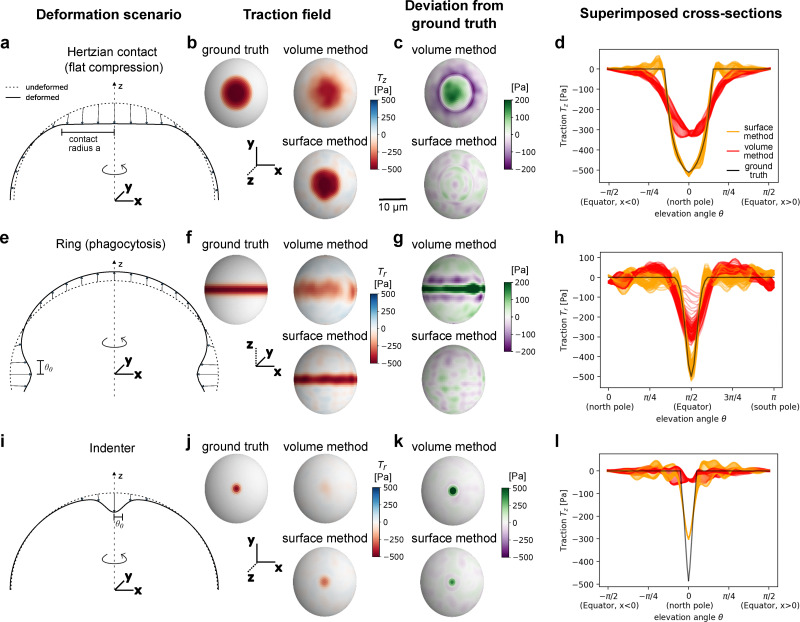
Traction reconstruction for three axisymmetric loading scenarios. (a)–(d) Hertzian contact (flat compression). (a) Top half of the deformation geometry, which is symmetric around the *xy*-plane for this and the following two scenarios. (b) Ground truth traction field of the principal traction component *T*_*z*_, followed by reconstructions obtained with the volume and surface methods. The surface method closely reproduces both the magnitude and spatial distribution of the reference traction. The volume method captures the overall structure. (c) Deviation and superimposed cross-sections (d) of the relevant traction component, showing that the volume method exhibits underestimation in the loaded region and compensating deviations in adjacent traction-free areas. (e)–(h) Ring (phagocytosis-like equatorial indentation). Again, both methods recover the overall profile shape of *T*_*r*_, with the surface method matching the ground truth more closely. (i)–(l) Indenter. For this strongly localized loading profile, the surface method recovers the spatial profile of *T*_*r*_ but underestimates peak magnitude. The volume method shows pronounced attenuation and spatial spreading of the applied traction. Full traction profiles are provided in Fig. S11–S13. Simulation parameters: *E*_0_ = 1500 Pa, *ν* = 0.4, *a* = 0.5 (Hertzian contact), *θ*^ring^_0_ = 0.1, *θ*^indenter^_0_ = 0.08, *T*_0_ = 500 Pa (ring and indenter).

To systematically compare the surface and volume methods, both were applied to simulated experimental data of three axisymmetric traction profiles, shown in Fig. 2. The first profile, known as Hertzian contact, mimics the compression of a sphere between two walls, replicating interactions with large bodies, such as other cells or rigid surfaces (Fig. 2(a)). The magnitude of indentation in the Hertzian contact profile is characterized by the contact radius *a*. The overall shape of the relevant traction component *T*_*z*_ is recovered successfully by both methods (Fig. 2(b)), even though the surface method matches the original profile more closely. As visible in both the deviation maps (Fig. 2(c)) and the super-imposed cross-section (Fig. 2(d)), the reconstruction of the volume method appears attenuated (∼30%) and spatially broadened, underestimating the peak magnitude and spreading significantly into the traction-free region. In contrast, the surface method exhibits a slight overshoot and ringing at the edge of the traction profile, likely due to the Hertzian contact's singular slope at the contact boundary, which is difficult to resolve with a finite-order spherical harmonic representation. The other two recovered traction components *T*_*x*_ and *T*_*y*_ are shown in Fig. S11.

The second profile is a Gaussian ring-like indentation, reproducing the compression of a sphere observed during phagocytic engulfment (Fig. 2(e)).^29^ The shape of the Gaussian is characterized by *θ*_0_ (width) and *T*_0_ (magnitude), see also Note S4. Again, the recovered *T*_*r*_ component of the volume method appears attenuated (∼30%) and spatially broadened, albeit less strongly than for the Hertzian contact (Fig. 2(f)–(h)). Interestingly, at the same time the volume method significantly overestimates the magnitude of the *T*_*θ*_ component (∼5-fold), and also shows spatial broadening for the component (Fig. S12). A potential explanation is that the underlying displacement field is already blurred in the volumetric reconstruction. Because the direct volume method obtains stresses by spatial differentiation of this field, the broadened displacement gradients can produce artificial angular shear, which manifests as a spurious *T*_*θ*_ component. For the surface method, *T*_*θ*_ is also overestimated, but to a lesser extent (∼3-fold) and without spatial broadening, likely because the surface-based regularization enforces smoother and more physically consistent traction fields.

The third profile reflects a Gaussian indentation, localized at the poles and designed to assess the reconstruction of point-like traction profiles as observed with focal adhesions or viral/particle entry (Fig. 2(i)). Again, the shape of the Gaussian is characterized by *θ*_0_ (width) and *T*_0_ (magnitude). For the width *θ*_0_ = 0.08 examined here, the shape of the volume method reconstruction is barely visible, but resembles the expected shape (Fig. 2(i)). The magnitude, however, is strongly attenuated (∼90%, Fig. 2(l)). For the surface method we also observe a significant attenuation (∼40%), which was not present for the other profiles, and an overestimation of the *T*_*θ*_-component (Fig. S12). The significant attenuation for the surface method can be attributed to the finite spatial resolution imposed by the maximum spherical harmonic order *l*_max_. When the width of the traction profile approaches the corresponding spatial sampling limit, the reconstruction amplitude decreases. This effect is explicitly shown in Fig. S14, where the performance of the surface method as a function of *θ*_0_ significantly drops below a certain width of the indenter.

The closed-form equations of all three traction profiles are shown in Note S4.^57,58^ The simulation and analysis parameters, described in Table S1, are based on experimental studies.^29,30^ The stiffness of the microparticles is characterized by Young's modulus *E*_0_ and Poisson's ratio *ν*. The set noise levels led to comparable variations in the vanishing traction components (see *T*_*x*_/*T*_*y*_ in Fig. S11 and *T*_*φ*_ in Fig. S12), enabling a fair comparison.

**Fig. 3:**
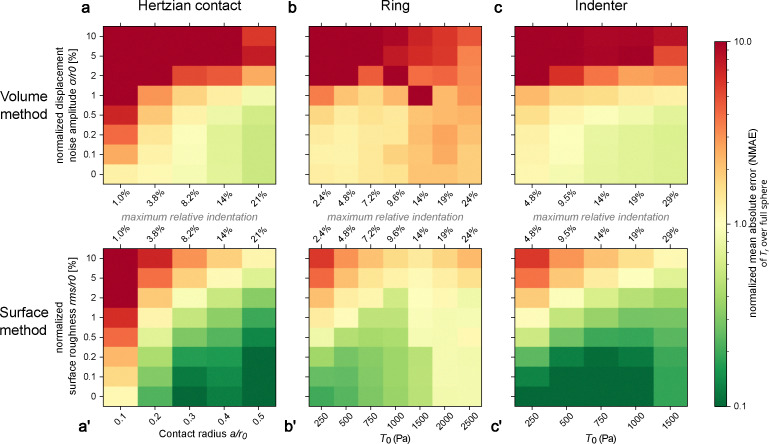
Reconstruction error scaling with displacement noise and loading strength. Normalized mean absolute error (NMAE, evaluated across the full sphere) of the radial traction field component *T*_*r*_ reconstructed by volume method (a)–(c) and surface method (a′)–(c′) for the Hertzian contact, ring, and indenter scenarios, evaluated over a range of noise levels and loading parameters. Given the chosen simulation parameters, 1% displacement noise corresponds to one pixel. An extended version of this figure is shown in Fig. S15. Traction profile parameters: *E*_0_ = 1500 Pa, *ν* = 0.4, *θ*^ring^_0_ = 0.1, *θ*^indenter^_0_ = 0.15, simulation parameters see Table S1.

We then numerically evaluated the errors across the sphere for a range of different noise amplitudes and indentation strengths using the normalized mean absolute error (NMAE) (Fig. 3, numeric error values see Fig. S15). Comparing both methods, we find that the errors for the surface method are consistently lower for all examined scenarios. As expected, the performance generally decreases with noise for both methods. Even though the absolute error values of the volume method are higher, the sensitivity of those errors to noise appears to be much lower. This indicates a robustness of the volume method to displacement noise in the microparticles.

The volume correlation algorithm appears to recover the field reliably, up to a certain noise level (nanoparticle displacement noise ∼1 pixel) where the errors diverge. The higher noise sensitivity of the surface method is expected as the surface roughness noise directly propagates into the measured surface displacements and therefore the corresponding traction. For the Hertzian contact profile, the errors of both methods decrease with larger indentation, minimum NMAE error values are 55% (volume) and 8% (surface), respectively, for a maximum relative indentation of 21%. For the ring profile, both methods show a significant decrease in performance with an increase in magnitude of the deformation, increasing from 20% to 87% (surface) and from 120% to 190% (volume) from the lowest to highest indentation considering no noise (maximum relative indentation ranging from 2.4% to 24%). For the indenter, lowest absolute NMAE values across all methods and scenarios of 3% were observed at 10–15% maximum relative indentations with the surface method considering no noise. Here, for the volume method, the lowest error values of 66% were observed at higher magnitudes around 30% indentation, again displaying a remarkable robustness to noise increase. The described trends did not change when the error evaluation was constrained to a localized region around the maximum force application (Fig. S16).

We also evaluated the aforementioned variant of the surface method where a lower number of spherical harmonics is used (*l*_max_ = 5), and only radial displacements are assumed.^31,32^ This corresponds to the initial guess before the optimization of *u⃑ via* the functional. This variation of the surface method shows higher errors than both volume and surface method in most cases apart from very high noise and indentation levels, as shown in Fig. S15. When the error evaluation was constrained to a region around the maximum force application (see Fig. S16), this “truncated” surface method outperformed the volume method, but both were again outperformed by the surface method. Following the discussion above and in Fig. S14, this can partially be explained by the lower number of spherical harmonic coefficients, which may not be able to sufficiently resolve the localized nature of the ring and the indenter (effective width ∼ 0.2/0.3 rad, minimum effective wavelength of Legendre polynomial ∼ 0.6). However, even for the more delocalized Hertzian contact, the errors of the truncated surface method are much higher (*e.g.*, 90% *versus* 9% of surface method at *a* = 0.3). This indicates that while computationally expensive, the optimization of the displacement solution *via* the energy functional appears to be the crucial step that gives the surface method its competitive edge. One may argue that this is caused by the significant additional information provided in the traction-free region. However, previous simulations using a scenario comparable to a Hertzian contact with *a* = 0.3 found that leaving out the traction-free region made almost no difference in performance in the magnitude range where the surface method significantly outperforms the truncated one.^29^ A possible explanation for why removing the information about the traction-free regions does not substantially affect the recovery is that the elastic energy and the traction-free penalty term have a similar potential landscape in the functional and minimize *u⃑* into similar directions. The elastic energy term appears to penalize non-physical displacement profiles. Accordingly, the assumption of the surface displacements being only radial (*u⃑*‖*e⃑*_*r*_) appears to not describe the physical reality of most systems, which is also apparent from considering the deformation sketches in Fig. 2(a), (e) and (i). Despite these shortcomings, there are regions of similar performance between surface method and truncated surface method, particularly at higher noise levels, where the truncated approach with significantly lower computational runtimes can be deemed sufficient.

### 3.2 Surface effects of volume method

To understand the substantial performance deficits of the volume method, we examined both its experimental and analysis hyperparameters. First, we systematically varied nanoparticle size and nanoparticle seeding density to assess whether this reduced reconstruction errors, with full results for each configuration given in Fig. S17. While we found that the error does depend on both parameters, and increased significantly especially for small nanoparticle diameters (50 nm) under the resolution limit, the parameters used throughout the simulations (200 nm diameter and 0.002 nanoparticles per voxel) are close to the optimal parameter choices for all scenarios and even the lowest error values obtained are still significantly above those of the surface method, showing that the experimental hyperparameters of the volume method are not responsible for the higher errors.

**Fig. 4:**
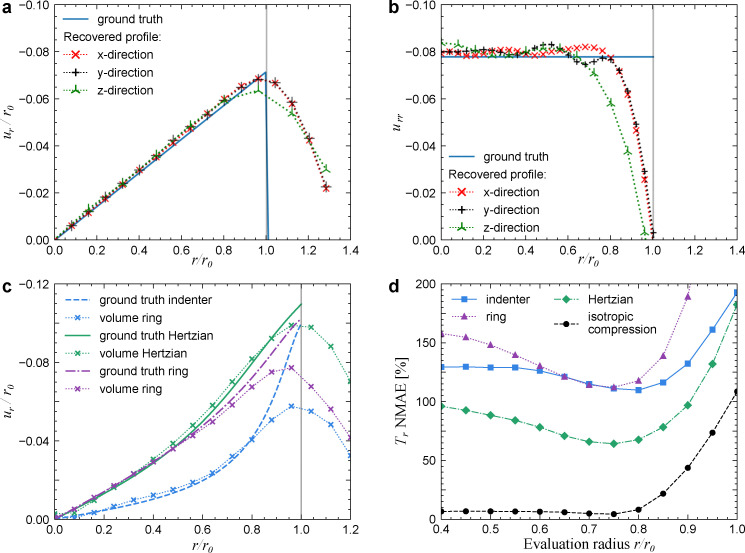
Surface effects of the volume method appear to be the main reason for the higher error rates. (a) Radial displacement *u*_*r*_ for isotropic compression profile. (b) Strain component *u*_*rr*_ = ∂_*r*_*u*_*r*_ (derivative of subfigure (a)) for isotropic compression profile, note that *T*_*r*_ ∼ *u*_*rr*_. (c) Radial displacement *u*_*r*_ for the previously examined scenarios, showing even larger deviations near the surface. (d) Normalized mean absolute errors for all scenarios, showing larger errors for localized profiles (ring, indenter).

Next, we examined the behavior of the volume correlation algorithm FIDVC^40^ close to the surface and its connection to the evaluation radius *r*_e_, an important analysis parameter. *r*_e_ defines the radius of the sphere on which the derivatives of the displacement field are evaluated to obtain the traction profile. In the original volume method publication,^30^*r*_e_ = 0.8*r*_0_ due to problems with the recovered displacement field directly at the surface. The problematic nature of these “surface effects” can be demonstrated when applying the volume method to the simplest deformation possible: the isotropic compression of a sphere under a given pressure, *i.e.* only a change in radius. Fig. 4(a) shows the values for the radial displacement *u*_*r*_ recovered with the FIDVC for the isotropic compression, which are very accurate up to *r*/*r*_0_ = 0.8 and then show slight deviations closer to the sphere surface (*r* = *r*_0_), with the absolute values still being reasonably close. However, to calculate the traction, we have to compute the strain *u*_*rr*_ and therefore the derivative ∂_*r*_*u*_*r*_. As a result, what at first sight appears to be a minor absolute deviation, leads to very strong relative deviations near the sphere surface, as shown in Fig. 4(b). We also observe stronger deviations in the *z*- than in the *x*- and *y*-directions, which we attribute to the lower resolution we set in that direction to mimic the lower resolution in that direction typical for *z*-stacks.

For the isotropic profile, *u*_*rr*_ and therefore also the traction is constant throughout the sphere due to the constant slope observed in Fig. 4(a). Therefore, decreasing *r*_e_ still leads to accurate results for this scenario. However, for more complex and especially more localized profiles, the slope changes significantly towards the sphere surface, shown in Fig. 4(c). The deviation of the FIDVC results from the ground truth is most prominent for the very localized indenter profile with the steep slope increase near the surface. Therefore, evaluating the components anywhere inside the sphere leads to significant underestimation in the traction profile for the volume method, as evident from Fig. 4(d). Regardless of evaluation radius, the obtained errors for the three examined scenarios are all more than a magnitude higher (60–200%) than that obtained for the isotropic compression (<10%). For this reason, the volume method is very suitable for measuring isotropic compression. In its standard configuration without a reference image, the surface method is unable to measure compression, as for the initial guess of the radial displacement solution it assumes an incompressible sphere. In theory, however, pressure could be measured by considering a reference picture of the microparticle and measuring the difference in total volume.

These findings point towards a central problem of the volume method: we can either move the evaluation radius inside to get a more accurate measurement of the displacement field, but lose the important information near the surface, or we move it further to the surface, but then lose accuracy because the volume correlation algorithm is not able to properly track displacements anymore due to the lack of nanoparticles close to and outside of the surface. The decomposition described in eqn (10)–(13) explains the underlying reason. When decomposing the traction on the surface *via* the Legendre polynomials, profiles that are more localized on the surface result in larger coefficient terms *a*_*n*_ and *e*_*n*_ for higher orders of *n*, corresponding to the necessity of higher frequency components to resolve the profile. These higher order terms then also result in higher powers of *r* for the displacement, considering the scaling of components ∼*a*_*n*_*r*^*n*−1^ and ∼*e*_*n*_*r*^*n*+1^. The higher powers *r*^*n*^ then lead to larger values near the surface of the sphere.

We also simulated blurring of nanoparticles by widening the PSF. Results are shown in Fig. S18. For both Hertzian contact and indenter scenarios, error increased with blurring strength and plateaued when the blur reached the order of 3 pixels. For the ring profile, surprisingly the blurring had only a limited impact on the reconstruction error. The effect, however, might be hidden in the generally higher baseline errors observed for the ring-loading configuration.

### 3.3 Experimental evaluation using DNA hydrogel microparticles

**Fig. 5:**
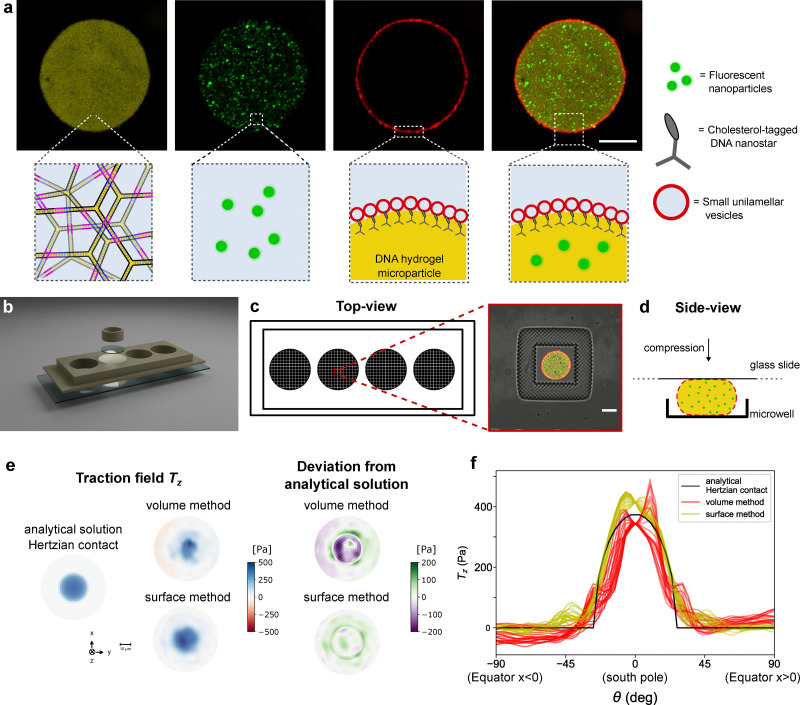
Design of experimental system and recovered traction profiles. (a) Confocal images (top row) and sketches (bottom row) illustrating the design and composition of DNA-HMPs engineered for this study. Confocal images show the DNA network composed of Cy3-labeled DNA nanostars (yellow, *λ*_ex_ = 561 nm) with embedded fluorescein-labeled nanoparticles (green, *λ*_ex_ = 488 nm) and coated with Atto-633-labeled SUVs (red, *λ*_ex_ = 640 nm). The final image shows a fluorescence overlay of all channels and components. The fluorescent nanoparticles thus label the volume; the DNA itself and the SUVs carry information about the surface shape, allowing for comparison of the methods on the same particle. Scale bar: 20 µm. (b) Exploded-view 3D rendering of the deformation setup as a custom 3D-printed 4-well holder (11 mm diameter wells), glued onto a glass slide and equipped with laser-printed PEGDA-based microwells of 40 µm diameter. Following DNA-HMP deposition into the microwells, the large wells were covered with a glass coverslide and the DNA-HMPs weighed down with a ring weight. (c) Sketch depicting the top-view of the observation chamber with the four large wells and printed microwells. The zoom image depicts a DNA-HMP deposited into one of the microwells. Scale bar: 20 µm. (d) Sketch depicting DNA-HMP deformation in a microwell by the deposited glass slide as a side-view. (e) Exemplary recovered traction profiles (bottom view on *T*_*z*_) for both methods from the same microparticle (assuming *E*_0_ = 1.1 kPa, *ν* = 0.4), and error/deviation from a Hertzian contact profile with a suitable contact radius (*a* = 0.45). (f) Superimposed cross-sections of traction profiles. Each line starts at the equator and finishes at the equator on the opposite side of the sphere.

Next we experimentally implemented the volume and surface methods to compare their performance in practice. For this purpose, we employed DNA hydrogel microparticles (DNA-HMPs), which enable both approaches within a single experimental system. As shown in Fig. 5(a), the cross-linked DNA forms a fully fluorescent sphere allowing us to recover the sphere surface needed for the surface method, while embedded nanoparticles facilitate the tracking necessary for the volume method. We additionally decorated the DNA-HMP surface with small unilamellar vesicles (SUVs) in an effort to more directly label the DNA-HMP surface. Reconstruction of the DNA-HMP surface from the SUV-coating images, however, proved unsuccessful for our setup (Fig. S10). Nonetheless, SUV-addition remains attractive for future experimental settings *in vitro* as the SUVs can be used to functionalize the DNA-HMP surface with ligands or receptors, forming more natural interfaces with cells. We also tuned DNA-HMP stiffness to *E*_0_ ≈ 1.1 kPa (Fig. S7), which is comparable to values utilized in previous studies (∼1.4 kPa^30^).

To apply controlled forces, DNA-HMPs were trapped in microwells and compressed from above by a glass coverslide with increasing load facilitated by the addition of a 3D-printed weight (see Section 2.7). This way, it was possible to lower the applied force again after deformation by removing the weight (Fig. 5(b)–(d)). As the glass slides are significantly stiffer (*E* ∼ GPa) than the DNA-HMPs, the particle is expected to deform with a Hertzian contact profile as introduced above in Fig. 2(a). The localization of microparticles in individual wells enabled us to take a reference picture of the uncompressed DNA-HMP for the volume method before or after compression.

We then applied the volume and surface method workflows established for the simulations to experimental data. Since our DNA-HMPs did not have a suitable indicator for traction-free regions for the surface method, this term does not contribute to the energy functional here, but should also not decrease performance significantly for the expected deformation levels.^29^Fig. 5(e) and (f) show our results for one representative example. We note that the obtained traction profiles for the bottom side of the particles facing the microwell are similar in shape to the analytical Hertzian contact solution for a suitable contact radius (with *a* = 0.45). Evaluating the NMAE from the analytical solution across the bottom half of the sphere (*z* < 0) yields 35% for the surface method and 64% for the volume method, again showing a performance advantage of the surface method. However, the volume method appears to perform much closer to the optimum NMAE achieved in the simulations without noise (56% for *a* = 0.5) than that of the surface method (8% for *a* = 0.45, both see Fig. S15). We attribute this to the aforementioned robustness to noise of the volume method, where the NMAE is basically unaffected up to displacement noise values of 0.5% px^−1^ in the simulations. For the surface method, on the other hand, as shown in Fig. 3, the NMAE strongly scales with surface noise, and assuming no other noise sources, the experimentally obtained NMAE for the given indentation (*a* = 0.45) suggests an effective surface roughness of around 2% for the surface method. Analyzing the surface roughness of the undeformed microparticle *via* the NRMS, we indeed find a value of ∼2.6% for the bottom half of the sphere.

For the top side of the DNA-HMP, the traction reconstructions by both methods appear less accurate (Fig. S20), reflected in higher NMAE errors of 130% for the volume method and 82% for the surface method. We attribute the decrease in performance to a deterioration of resolution near the top of the surface, as evident by the recovered microparticle surface (Fig. S21). The top part clearly shows visible roughness with an NRMS value of 3.3% (Fig. S19), which again directly translates into higher NMAE for the surface method. As the performance of the volume method appears to be affected as well, we assume a decrease in imaging quality (fluorescence signal compared to noise) towards the top, possibly caused by higher scattering, to be responsible for the performance decrease. This problem is common for *z*-stack imaging and has also been observed for polystyrene particles, inspiring the usage of deformable acrylamide-*co*-acrylic acid microparticles in the original surface method study.^29^ The particles used in that study were also more homogeneously spherical, with an NRMS of <1%, which as in our case includes sphericity deviations caused by both imaging and variations in the particles themselves. The asymmetry could partially also be caused by the glass slide on the top, while it is pushed down by the weight, or by movements of the scanning microscope. Further, because our force application occurred along the *z*-axis, along which resolution is typically lower in confocal microscopy, results might improve when considering a different axis for force application. Our results imply that with improved imaging and force control, reconstruction quality could be higher in future experimental setups. Three-dimensional microscopy images for this DNA-HMP are shown in Fig. S22. Images of a second microparticle are given in Fig. S23, showing again a spatial broadening of the traction profile for the volume method and similar problems on the top side of the microparticle compression setup.

Experimental implementation of the volume method proved challenging, mainly due to the simultaneous requirements of controlled force application, high-quality volumetric imaging, and stable reference image acquisition. Consequently, the number of microparticles which yielded reliable reconstructions was limited. For the surface method, deformed surface shapes could be reconstructed and traction profiles computed for more examined microparticles, again highlighting its broader applicability due to ease of experimental implementation. Additional microparticle profiles showing surface method evaluations are provided in Fig. S24. The traction fields mostly reflect the compression, but they also reveal that improved resolution and force application are necessary to cleanly resolve the expected Hertzian contact profile, particularly given that the surface method relies only on information about the deformed surface shape, which has to be captured at high quality. Despite sample size limitations, our experimental observations are consistent with the trends observed in our simulations: the volume method exhibits attenuation and spatial broadening in the reconstructed traction profile, while the surface method generally produces sharper reconstructions but remains more sensitive to imaging noise.

### 3.4 Comparison with nonlinear elasticity models

**Fig. 6:**
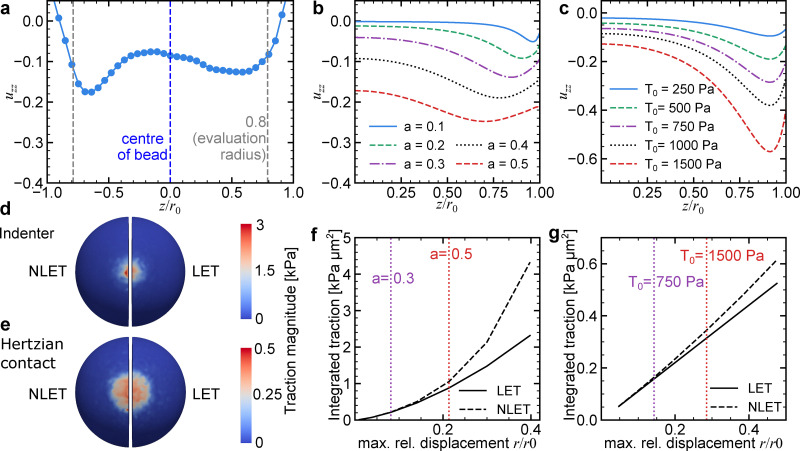
Limits of linear elasticity. (a) Experimentally obtained strain values of the full sized image along the *z*-axis. The evaluation radius is indicated by the dashed vertical lines. Same data as in Fig. 5. (b) Analytical strain solution (*u*_*zz*_) for Hertzian contact scenario of different compression magnitudes, characterized by the contact radius *a*. Note that now we show only one half due to symmetry. The curves have the same shapes as the experimental data. (c) Analytical strain solution for the Gaussian indenter, compression characterized by maximum traction *T*. Here, the maximum observed strains are generally higher near the surface, but increase towards the center of the sphere, in line with the observations in Fig. 4. (d) Gaussian indenter of high magnitude (maximum relative indentation 29%), side-by-side comparison of traction profile recovered *via* FEM using non-linear elasticity theory (NLET, left) and linear elasticity theory (LET, right). Both yield similar results. (e) Side-by-side comparison of LET *vs.* NLET for Hertzian contact (maximum relative indentation 8%), again showing similar results. (f) Integrated traction magnitude for Hertzian contact of FEM simulations, showing good agreement between LET and NLET up to contact radius *a* = 0.5. (g) Integrated traction magnitude for indenter scenario, showing good agreement even at high compression.

Some of the simulations performed represent strong compression scenarios ranging up to 30% in maximum relative indentation. The maximum relative displacement in the experiments are estimated to be up to 20%. This raises the question if the assumptions using linear elasticity theory (LET) still hold. LET is generally considered to be valid for strains up to *u*_*ij*_ ≲ 0.1. In Fig. 6(a), we reconstruct the strains of the experimental data used in Fig. 5. One sees that there is a typical compressive strain around 0.1 in the middle of the microparticle, which then reaches peak values of up to 0.2 closer to the surface, until going down to 0.0 at the two surfaces. We also note that the two sides are not equivalent, because the microparticles are placed on the bottom surface and loaded from the top.

The experimentally measured curve agrees well with the simulation results for the Hertzian contact shown in Fig. 6(b), which by construction are symmetric in bottom and top, thus we only show one side. For the indenter scenario, the strains observed in the simulations range even higher, up to 0.6 for the maximum indentation level (Fig. 6(c), *T* = 1500 Pa).

To test if the assumption of linear elasticity leads to errors in the traction estimation, we performed finite element method (FEM) simulations comparing LET and NLET, the latter implemented by a hyperelastic (neo-Hookean) model. As shown in Fig. 6(d)–(g), LET and NLET yield similar results up to a maximal relative displacement of 0.2, which is the level reached in our experiments. The experimentally estimated contact radius (*a* = 0.45) is just below the limit of when the models start to diverge for the Hertzian contact (Fig. 6(f)), showing that even though the strain magnitude exceeds 0.1, the assumptions of LET still seem to hold. For the indenter scenario, the agreement seems to persist even to higher levels (Fig. 6(g)).

## 4 Conclusions

In this work, we performed a systematic comparison of the two most common strategies used in MP-TFM: the volume method and the surface method. Using simulated traction profiles representing three biologically relevant loading scenarios, we found that considering similar noise levels, the surface method consistently reconstructs traction fields with substantially lower errors than the volume method. Although it might appear difficult to compare two methods that are very different in nature, we note that both can be considered to be regularized and that both have been optimized for MP-TFM.

Analysis of the displacement fields revealed that this performance difference originates primarily from surface artifacts inherent to the digital volume correlation algorithm used in the volume method. While the recovered displacement field remains accurate throughout most of the particle interior, tracking accuracy deteriorates close to the particle surface due to the lack of fiducial markers outside the particle. Because traction reconstruction requires spatial derivatives of the displacement field, these small deviations become strongly amplified near the boundary. Consequently, traction fields must be evaluated at radii further inside the particle, which leads to a loss of information near the deformed surface where the forces are applied.

We further provide quantitative error estimates across a range of noise levels and loading strengths for the volume method, the surface method, and a simplified “truncated” surface approach that omits displacement optimization, offering faster computation at the expense of accuracy. These results establish practical benchmarks for experimental MP-TFM studies. Our analysis shows that the volume and surface methods exhibit different sensitivities to measurement noise. The surface method achieves significantly lower reconstruction errors under low-noise conditions but shows a strong dependence on surface roughness. In contrast, the volume method is substantially more robust to displacement noise of the embedded nanoparticles because the correlation algorithm averages information from many markers within the particle volume. As a result, the performance gap between the two methods decreases at high surface roughness noise levels, making the surface method more sensitive to imaging conditions and to the sphericity of the used microparticles. For future applications, it is therefore advisable to improve the performance of the surface method by establishing more accurate estimates of the reference configuration. It is left to future work to decide which method performs best in the context of complex and time-dependent processes in real tissues, for which confounding effects like scattering have to be taken into account. Here it might be interesting to develop new pipelines, like an inverse regularized method for the volume method, especially in light of its success in classical 2D-TFM.

To experimentally validate our findings, we implemented both approaches using DNA-HMPs equipped with fluorescent surface labels and embedded nanoparticles. Compression of the particles in a custom microwell setup produced traction profiles consistent with a Hertzian contact model, and allowed for reconstruction by both methods. In agreement with the simulations, the surface method yielded lower reconstruction errors, although the difference between the two approaches was reduced under experimental conditions due to imaging noise and the lower noise sensitivity of the volume method. The lower noise sensitivity partially stems from the necessity of the reference image for the volume method, but generally both methods profit from high-quality microscope images. Still, because the surface method does not need a reference image, it proved easier to apply reliably, as it is independent of image alignment or unintended nanoparticle motion, which could occur due to microparticle rotation. As our experimental analysis was limited to the Hertzian contact scenario, more experimental work is needed to verify our simulation predictions for the ring and indenter scenario, even though it might be difficult to obtain a ground truth in these cases. Another interesting avenue of investigation is the use of viscoelastic materials.

Beyond the method comparison, our results demonstrate that DNA-HMPs provide a versatile platform for MP-TFM experiments. Their tunable mechanical properties and compatibility with multiple fluorescent labels enable simultaneous implementation of both traction reconstruction strategies. The lipid SUV coating enables further biological functionalization, *e.g.*, with membrane proteins like E-cadherins and thus the creation of more cell-like interfaces, which may facilitate future studies of cellular force generation.

Overall, our results suggest that even though the volume method in principle relies on fewer assumptions and uses more information, namely the reference image and the full displacement field, the surface method is generally the preferred approach for MP-TFM due to its higher reconstruction accuracy and simpler experimental implementation. However, the volume method may remain advantageous in situations where surface imaging is compromised, such as near optical interfaces or in strongly scattering environments. Our findings provide practical guidance for selecting and interpreting traction reconstruction methods in future MP-TFM studies.

## Author contributions

S. B., B. K. K., T. W., C. M., and T. A. performed the research. S. B., B. K. K., and C. M. wrote the computer code and performed the data analysis. B. K. K., T. W., and T. A. performed the experiments. S. B. wrote the original draft of the manuscript, supported by T. W. K. G. and U. S. S. conceptualized and supervised the research. All authors reviewed the manuscript.

## Conflicts of interest

There are no conflicts to declare.

## Supplementary Material

SM-022-D6SM00242K-s001

## Data Availability

The supplementary information (SI) file contains Fig. S1–S24, Tables S1 and S2, and Notes S1–S4. See DOI: https://doi.org/10.1039/d6sm00242k. We provide a Python script and necessary files to run simulations of a Hertzian contact scenario with evaluations by the volume method and the surface method, a JupyterLab notebook for surface method reconstructions of deformed DNA-HMPs, and a JupyterLab notebook supplemented by exemplary displacement data and results for the FEM comparison of linear elasticity and hyperelasticity, on GitHub (https://github.com/brewburgr/MPTFM). The experimental data is available at https://doi.org/10.11588/DATA/KWOD5A.
