# Supplementary material for: Volume and surface methods for microparticle traction force microscopy: a computational and experimental comparison
Source: Soft Matter. 2026 Jun 17;22(30):4978–93. doi: 10.1039/d6sm00242k (PMC13288728; doi:10.1039/d6sm00242k)
Supplement: SM-022-D6SM00242K-s001 [file SM-022-D6SM00242K-s001.pdf]

Supplementary Information (SI) for Soft Matter.
This journal is © The Royal Society of Chemistry 2026

Supplementary information (SI)

Volume and surface methods for microparticle traction force microscopy: a computational and experimental comparison

Simon Brauburger, Bastian K. Kraus, Tobias Walther, Cornelis Mense, Tobias Abele, Kerstin Göpfrich and Ulrich S. Schwarz

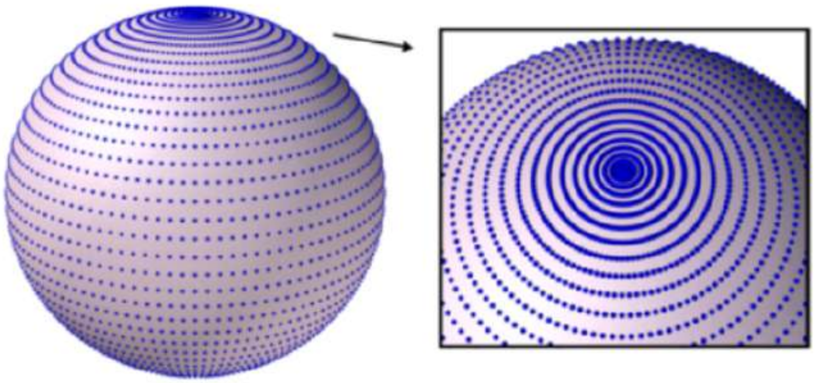

Figure S1 Visualization of a Gauss Legendre quadrature (GLQ) mesh.[51] This mesh is used for the minimization and traction evaluation in the surface method, and it allows for an efficient workflow together with the spherical harmonics (SH) approach.[29,36]

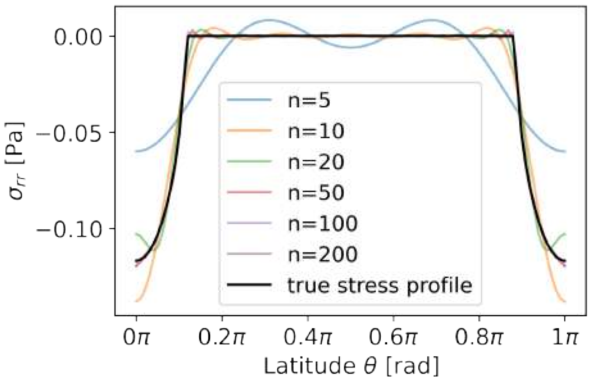

(a)

$n_{\max}$	NMAE [%]
5	74.9
10	20.0
20	10.0
50	3.1
100	2.0
200	0.5
500	0.1
1000	0.04

(b)

Figure S2 Decomposition accuracy test. (a): True and reconstructed stress profiles σ_{rr} for a Hertzian contact scenario. The cutoff $n = n_{\max}$ is the maximum sum order used in the decomposition of the analytical solution (see Eq.(10)[44]). (b): Normalized mean average error (NMAE) for different $n_{\max}$. For $n \geq 50$, the deviations are sufficiently small in comparison to the errors expected by the methods themselves. We choose a cutoff $n_{\max} = 50$, which provides an acceptable 3% deviation for the Hertzian contact profile at reasonable runtimes for the simulation of the experimental data ($\mathcal{O}(1\,\mathrm{min})$). As the Hertzian contact profile has a sharp edge, which requires more higher order terms to be resolved accurately, we can expect the results for smoother profiles, e.g., the Gaussian indenter or ring, to be even more accurate.

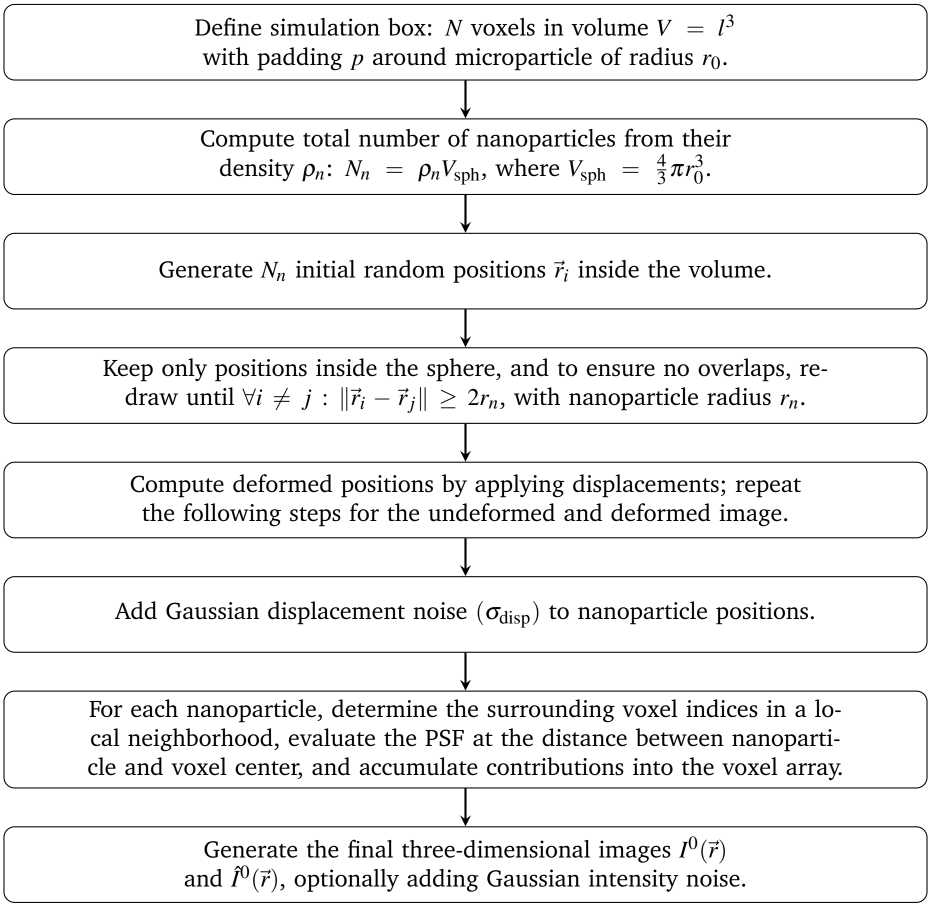

Figure S3 Workflow for generating synthetic reference and deformed three-dimensional nanoparticle images for the volume method. Steps include volume definition, nanoparticle placement with minimum distance and inside the microparticle, displacement application, Gaussian displacement noise, and PSF-based intensity computation. The redrawing in step 4 is computationally expensive as the distance matrix to all other points needs to be calculated, and could be skipped, allowing for nanoparticle overlap. For standard parameters, given in Table S1, the runtime is in the range of seconds.

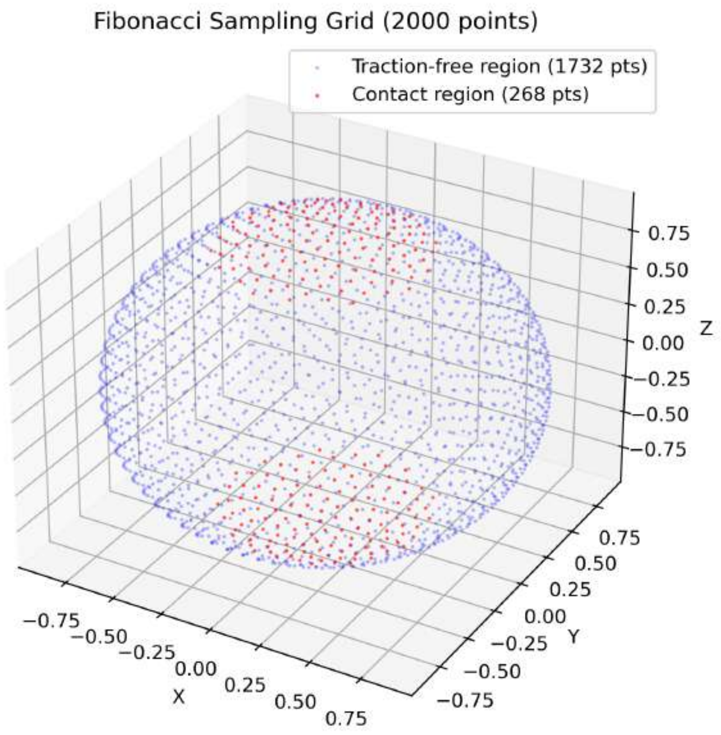

Figure S4 Visualization of a (deformed) Fibonacci grid, exemplary synthetic data for the surface method workflow. The set of points describing the microparticle surface is used to simulate a Hertzian contact ($a = 0.5$) with $N = 2000$ Fibonacci samples (here normalized to a unit sphere), and shown is the polar area of force application (red) as well as the region known to be traction-free (by the prescribed traction profile, see Note S4) used for the functional minimization (blue). Sampling on a Fibonacci grid leads to almost even spacing between the points, and the oversampling at the poles that a Gauss-Legendre quadrature (GLQ) would introduce is prevented. This oversampling would be advantageous for Hertzian contact and indenter profiles with force application at the poles, but disadvantageous for ring profiles, where force is applied in the equatorial region.

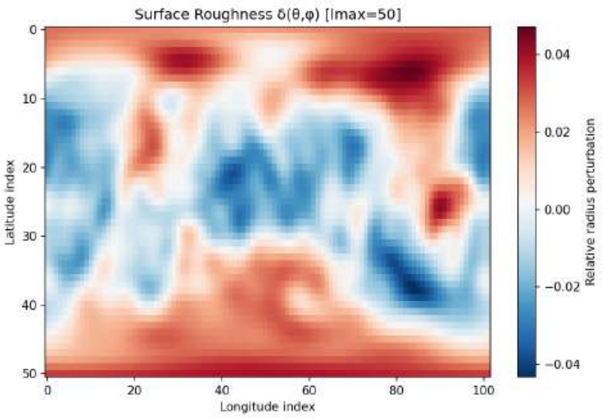

Figure S5 Surface roughness noise generation for surface method simulations. Visualization of an exemplary surface roughness distribution $\delta(\theta,\varphi)$, implementing radial perturbations with a spectral approach, here with a prescribed root-mean-square (rms) of 0.02 (relative to the reference radius r_0). This approach is used to simulate noise for the surface method as surface roughness, which can be quantified compactly by the rms. Indices cover the sphere but do not reflect degree or radian units.

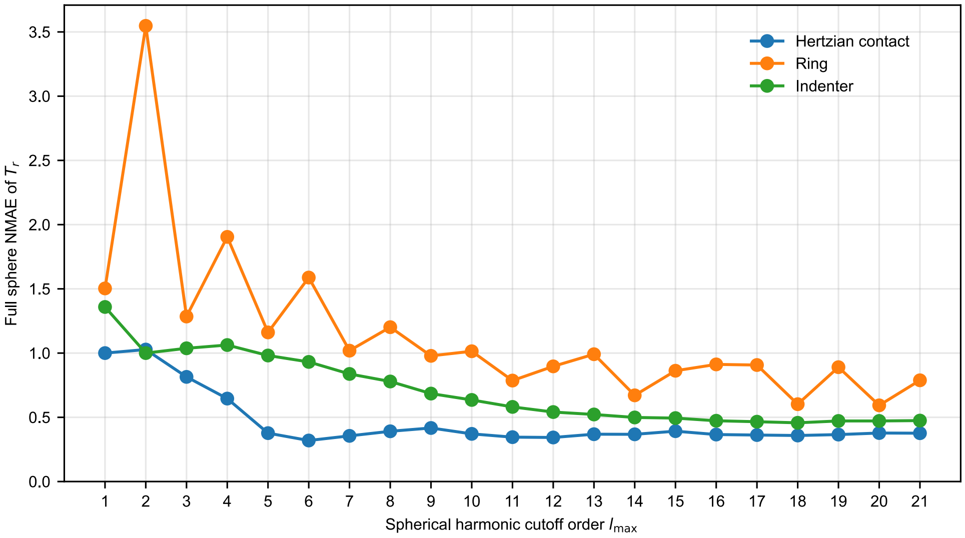

(a) NMAE full sphere.

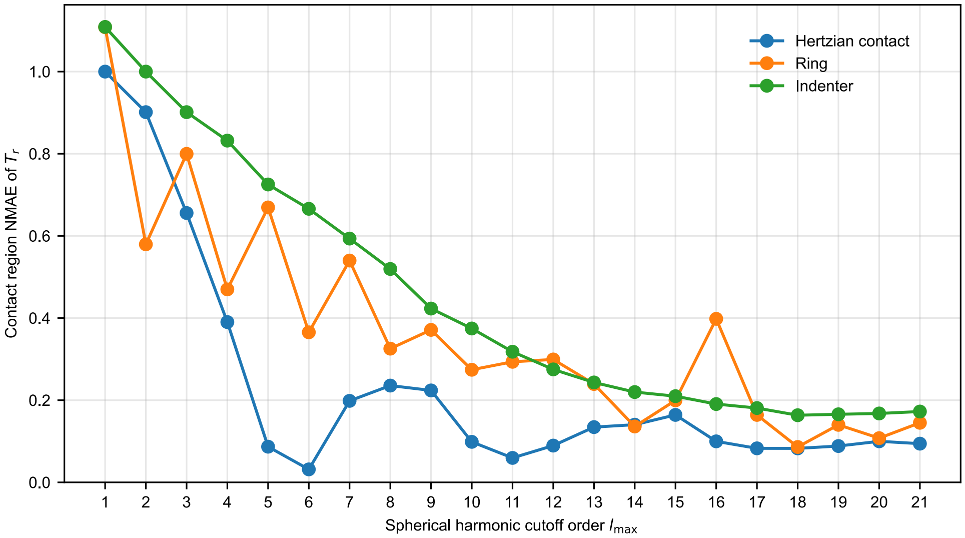

(b) NMAE contact region.

Figure S6 Surface method spatial resolution analysis. We evaluated traction reconstructions with respect to the spherical harmonic cutoff order $l_{\max}$ for the minimization, evaluated either over the full sphere (a) or within the contact region only (b). The normalized mean absolute error (NMAE) of the reconstructed traction field component T_r is shown as a function of harmonic cutoff order for the examined deformation scenarios (Hertzian contact, ring, indenter). Reconstruction accuracy improves overall with increasing harmonic order, with errors decreasing rapidly at low $l_{\max}$ before approaching a plateau around $l_{\max} \approx 18$. Beyond $l_{\max} > 20$, only marginal accuracy improvements are observed. At the same time, computational cost increases, scaling approximately with $l_{\max}^2$. These findings motivate the use of $l_{\max} = 20$ as the standard reconstruction parameter throughout this work, providing a suitable balance between reconstruction accuracy and computational efficiency . Less localized traction profiles, such as Hertzian contact, converge at lower harmonic orders ($l_{\max} \approx 15$), whereas highly localized traction distributions, such as the indenter geometry, require higher cutoffs to resolve sharper spatial features. More localized experimental force distributions may require higher harmonic orders together with correspondingly high-resolution surface reconstructions to avoid overfitting and aliasing (see also Fig. S14). Characteristic Gibbs-like oscillations are observed near the ring equatorial edge at low harmonic orders and diminish with increasing $l_{\max}$, illustrating why localized traction profiles require higher harmonic resolution than smoother force distributions.

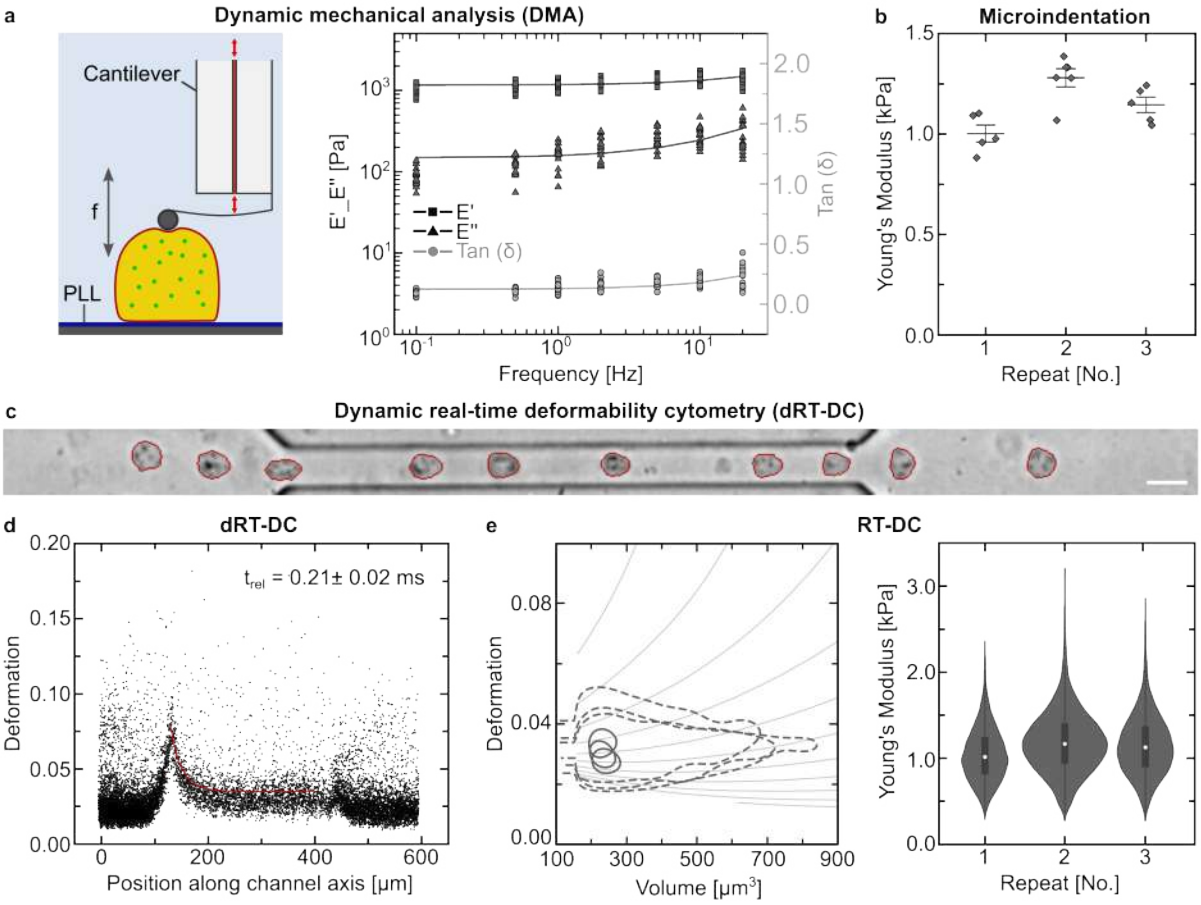

Figure S7 Mechanical characterization of DNA-HMPs for MP-TFM. (a) Schematic outlining DNA-HMP analysis via dynamic mechanical analysis (DMA) with DNA-HMPs attached to a glass substrate using electrostatic interaction following PLL-coating of the glass slide. Graph depicting DMA data of the DNA-HMPs. The storage modulus E' (squares) and the loss modulus E'' (triangles) are plotted on a logarithmic scale, $\tan(\delta)$ (E''/E', filled circles) is plotted on a linear scale. (b) Analysis of Young's modulus of the DNA-HMPs as extracted from Hertzian-fits to microindentation curves on the DNA-HMPs. The data is depicted as individual data points for each of the triplicate measurements also showing the mean ± standard deviation. (c) Composite image of the DNA-HMPs in the flow channel during dynamic real-time deformability cytometry (dRT-DC). The DNA-HMPs are initially spherical prior to entering the flow channel and get deformed strongly upon insertion into the channel. Traversing the channel, the DNA-HMPs relax into a steady-state deformation and return to their spherical shape after leaving the channel. Scale bar: $20\,\mu$m. (d) Deformation over position along channel axis scatter plot of the DNA-HMPs during dRT-DC. Each point corresponds to an individual DNA-HMP (n = 13000). The relaxation time t_{rel} of the DNA-HMPs was extracted from the exponential fit of the relaxation scatter plot (red curve) as previously described.[38] (e) Analysis of DNA-HMPs via real-time deformability cytometry (RT-DC). Contour plot depicting DNA-HMP steady-state deformation over particle volume for triplicate measurements showing the 50th percentile (dashed line) and 95th percentile (solid line). Isoelasticity lines derived from numerical simulations are shown additionally, indicating stiffness changes where a steeper slope corresponds to softer particles. The data depict strong overlap and thus good reproducibility between replicates. Young's modulus of the DNA-HMPs as derived from RT-DC steady-state measurements. Plot depicting the measured Young's modulus of n = 3 replicates of the DNA-HMPs (n_1 = 8913, n_2 = 16021, n_3 = 10635). The data is presented as violin plots showing the median value (white dot) of each measurement with boxplots encompassing the 25 - 75 % percentiles and a whisker length of 1.5 IQR. Mechanical analysis of the DNA-HMPs was conducted via dynamic mechanical analysis (DMA) by microindentation as well as real-time deformability cytometry (RT-DC) as outlined in a previous publication.[38] The DNA-HMPs were adhered to a glass substrate following electrostatic interaction with poly-l-lysine (PLL, MW = 150 kDa - 300 kDa) and measured with increasing indentation frequency using a spherical cantilever tip (Fig. S7a). DMA revealed largely frequency-independent storage moduli E' for the DNA-HMPs used in this study. Further, E'' remained below E' for all tested frequencies with the loss tangent $\tan(\delta)$ staying below 0.3 even at the highest frequency tested ($20\,\mathrm{Hz}$). This denotes the DNA-HMPs used here as behaving predominantly elastic. The mean Young's modulus E of the DNA-HMPs across triplicate measurements was further measured at $(1142 \pm 169)\,\mathrm{Pa}$ (Fig. S7b). Additionally, we performed dynamic real-time deformability cytometry (dRT-DC) at $0.04\,\mu\mathrm{L/s}$ total flow rate on the DNA-HMPs. As shown in Fig. S7c/d, deformation of the particles peaked upon channel entry, showing quick relaxation of the DNA-HMPs into a bullet-shaped steady-state deformation around 0.035 (Fig. S7c/d/e). An exponential fit to the slope of DNA-HMP relaxation scatter plot following initial deformation then revealed a characteristic response time τ of $(0.21 \pm 0.02)\,\mathrm{ms}$. Further, extraction of the Young's modulus E of the DNA-HMPs as measured via RT-DC at $0.04\,\mu\mathrm{L/s}$ total flow rate showed a mean value of $(1117 \pm 720)\,\mathrm{Pa}$ across the three triplicates and thus good agreement with the microindentation data (Fig. S7e). Calculation of the apparent viscosity of the DNA-HMPs used here following $\tau = \eta / E$[38] showed a low viscosity of $0.245\,\mathrm{Pa \cdot s}$, underlining the predominantly-elastic behavior of the DNA-HMPs. The marked increase in stiffness of the DNA-HMPs used in this study compared to their non-modified counterparts reported earlier[38] can be explained by the addition of the fluorescent nanoparticles and SUV-coating; both of which can be expected to result in a stiffer network, yet do not seem to impair overall network flexibility.

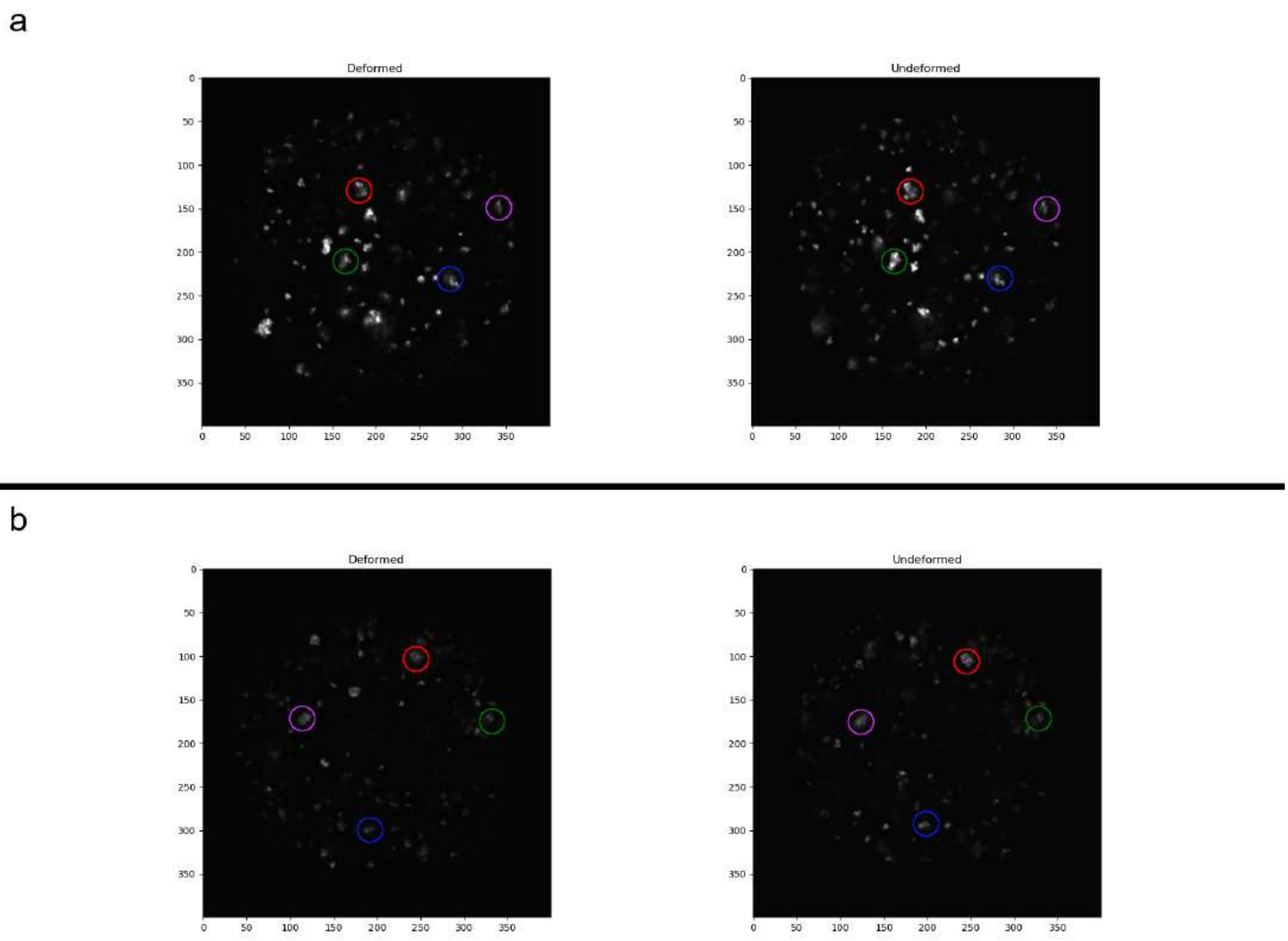

Figure S8 Visualization of preprocessed nanoparticle microscope images for the volume method at two different representative heights in the z-stack, consisting of 157 slices total. Left-hand side: Deformed configuration. Right-hand side: Undeformed (reference) configuration. (a) $z = 65$. (b) $z = 105$. In these aligned images, it is noticeable how several distinct nanoparticles moved from the undeformed to the deformed configuration, some of which are marked by colored circles. Units are voxels. The microparticle is the same one as in Fig.S9 and Fig.S10, and was analyzed in Fig.5(e, f). Preprocessing for the volume method. Here, the microscope images of the nanoparticles inside the DNA-HMPs in reference and deformed states are first preprocessed to allow for accurate correlation tracking. A Python pipeline has been developed, which first converts the nanoparticle images from .czi to .mat files, suitable for the correlation tracking algorithm (FIDVC), which runs in Matlab.[40] Special care must be taken with the coordinate ordering, as microscope images are typically saved as arrays with coordinate order (z, y, x), while Python programs usually employ (x, y, z), and Matlab uses (y, x, z). The workflow starts by loading in two files (reference and deformed), and optionally padding them symmetrically with zero intensity stacks along the z-dimension. This can be done because typically the deformed microparticle image will contain fewer z-stacks than the undeformed one because it will be compressed along the z-axis, even though during microscopy, a few extra z-stacks are captured above and below the microparticle to ensure it is fully imaged. Usually the smaller value of the two z-dimensions between the two images is chosen as the objective z-dimension after cropping, but especially for larger deformations, the padding step is important as to not cut off the reference microparticle with fewer z-stacks later, which would lose information. In these cases, adding 10-20 voxels in z as a buffer could improve results for the present work. Then, a defined microparticle intensity threshold is used for a boolean mask of voxels assumed to be part of the physical microparticle, approximating its shape. Next, the intensity-weighted center of mass of both microparticles is computed in three dimensions, employing the mask. Bounding boxes are calculated by generating start and end indices centered on the center of mass, but clamped to stay inside the volume. The objective size in the x- and y-dimension is defined as 400 voxels each for the present work, which is typically sufficient to capture the entire microparticle. Because the center of masses might be different, the resulting windows can slightly differ. To ensure the dimensions of the resulting images match exactly, the crop size is defined as the minimum overlapping size in each dimension, reducing both bounding boxes to the same shapes. Finally, both volumes are cropped by the computed and clamped indices. An appropriate threshold is applied to suppress background noise while not losing information, and images are saved as .mat files for correlation tracking. Visualizations of the images throughout the z-stacks as well as intensity histograms are plotted throughout to check intermediary and final results, and tune parameters. It was found that the tracking algorithm and recovered displacements depend sensitively on properly aligned images. The two z-stacks of aligned images that are visualized show that singular nanoparticles can already be tracked by eye, and a meaningful displacement field for the volume method could be extracted by the FIDVC.

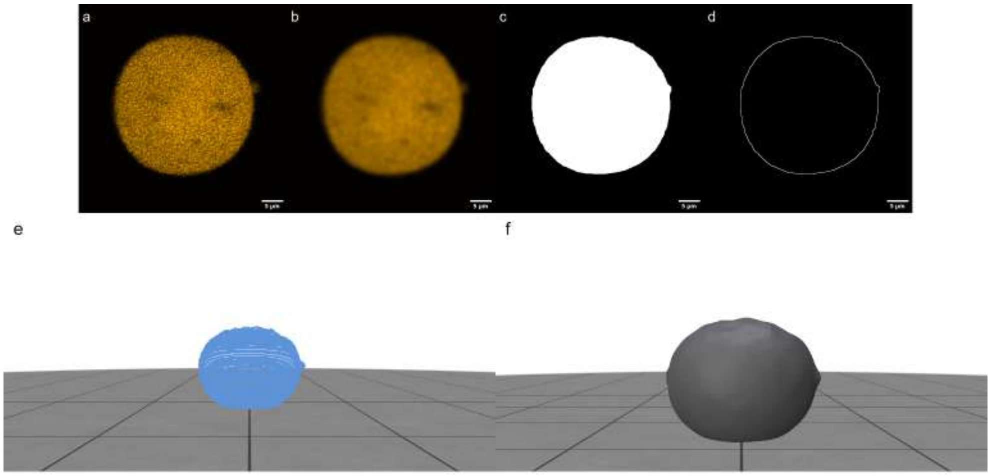

Figure S9 Preprocessing for the surface method, which requires the surface shape of a deformed microparticle. (a) Central z-stack of the original microscope image of the DNA network structure (Cy3). (b) A Gaussian blur is applied. (c) A threshold is applied by Otsu's method. (d) After filling holes, an outline can be computed. (e) Visualization of the resulting set of points describing the microparticle surface. (f) Reconstruction of the surface shape by Poisson's method after preprocessing and calculating normals. (a-d) are performed in Fiji[46] (scale bars $5\,\mu\mathrm{m}$). (e, f) are performed in GeoV.[47] The set of points can be saved as a .ply file. Analysis results for this microparticle are shown in Fig.5(e, f). Preprocessing for the surface method. Here, in order to analyze the DNA-HMPs, the data was preprocessed to yield a reconstructed DNA-HMP surface, using the fluorescent Cy3 labels in the DNA network to recover a set of points describing the microparticle surface. First, the acquired z-stacks were opened in ImageJ 1.54f (NIH[46]), filtered using a Gaussian blur filter ($\sigma = 4.0$), and threshold-adjusted using Otsu's method with parameters suitable for all z-stacks to yield binarized images. After that, the outline of the resulting binarized image stack was created after filling holes using Fiji's inbuilt functions, and saved as a .tif file. Surface reconstruction was then conducted using the software GeoV.[47] Within the software, the resulting set of 40,000 points was simplified to 0.4% world units and the data pre-cleaned to deplete outliers (0.5 probability). Surface normals were calculated with 2 smooth iterations, and the surface reconstructed using the screened Poisson method with a reconstruction depth of 5. All other parameters were kept as default. Parameters could be tuned in Fiji and GeoV, to yield smooth surface reconstructions while avoiding holes; though care must be taken not to falsify the observed surface shape. The resulting surface was then saved as a .ply file for deformation analysis with the surface method. The process is visualized for an example microparticle in Fig.S9, which also reflects differences in signal quality at the top and bottom of the microparticle.

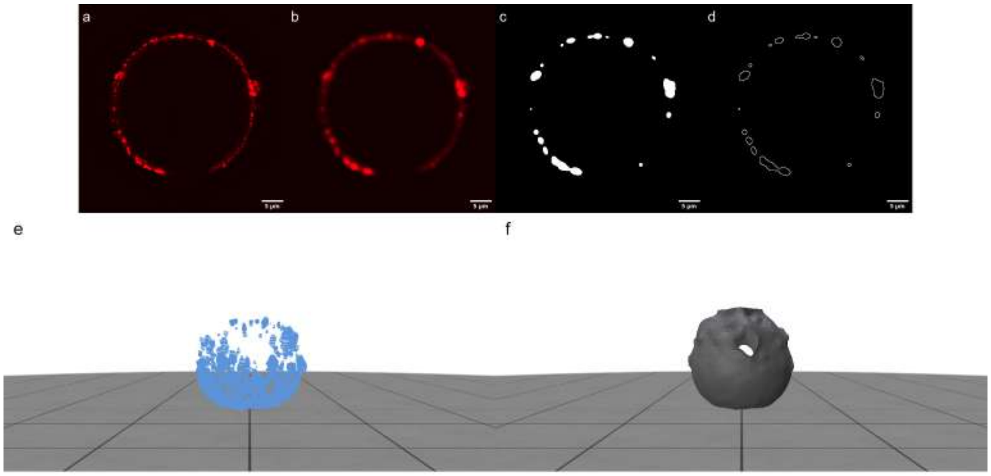

Figure S10 Attempted preprocessing of the surface label by the lipid SUV coating for the same microparticle as in Fig. S9. (a) Central z-stack of the original microscope image, showing the microparticle surface with small irregularities at high resolution. (b) A Gaussian blur is applied. (c) A threshold is applied by Otsu's method. (d) After filling holes, an outline can be computed, but the surface is not clearly resolved. (e) Visualization of the resulting set of points describing the microparticle surface, containing holes and missing points at the top due to the optical setup, which also led to problems defining consistent thresholds. (f) Reconstruction of the surface shape by Poisson's method from the set of points after preprocessing and calculating normals. (a-d) are performed in Fiji[46] (scale bars $5\,\mu$m). (e, f) are performed in GeoV.[47] The irregular surface containing holes is problematic, and it is not simple to smooth it out over all z-stacks. It does not reflect the deformed microparticle shape and cannot be used for the surface method. There have also been attempts to analyze the SUV coating (red), which was meant to serve as a surface indicator and could be useful in further biological setups, similar to the antibody coating for the traction-free region in the original publication.[29] However, the surface could not be smoothly reconstructed this way. The main issue seems to lie in the fact that the resolution of the employed microscope is too high, leading to small irregularities and holes in the surface reconstruction, which were difficult to smooth out consistently over all z-stacks; moreover, the optical setup and photobleaching affected the reconstruction. The process is visualized for an example microparticle in Fig. S10. However, as Fig. S9 shows, reconstructions are possible using the Cy3 labels.

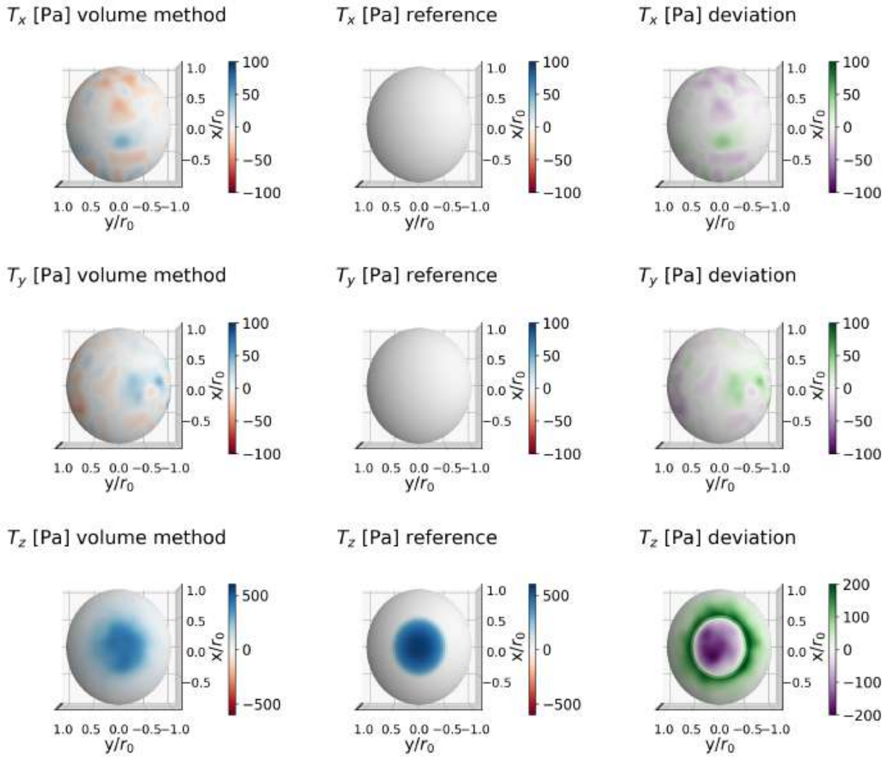

(a) Volume method.

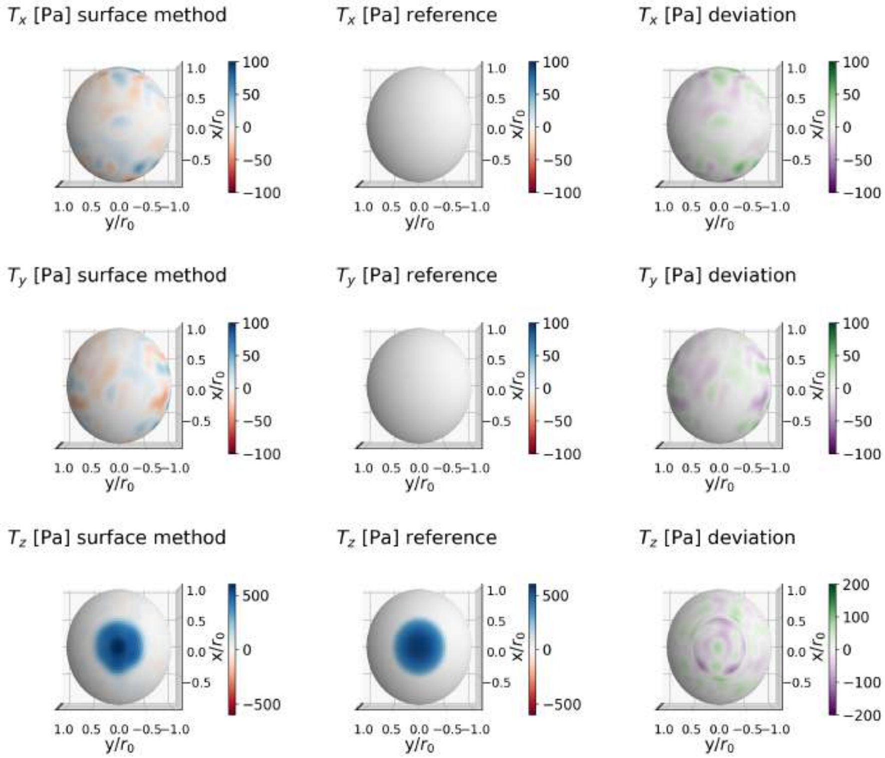

(b) Surface method.

Figure S11 Full simulation results of all three traction components for the Hertzian contact scenario.

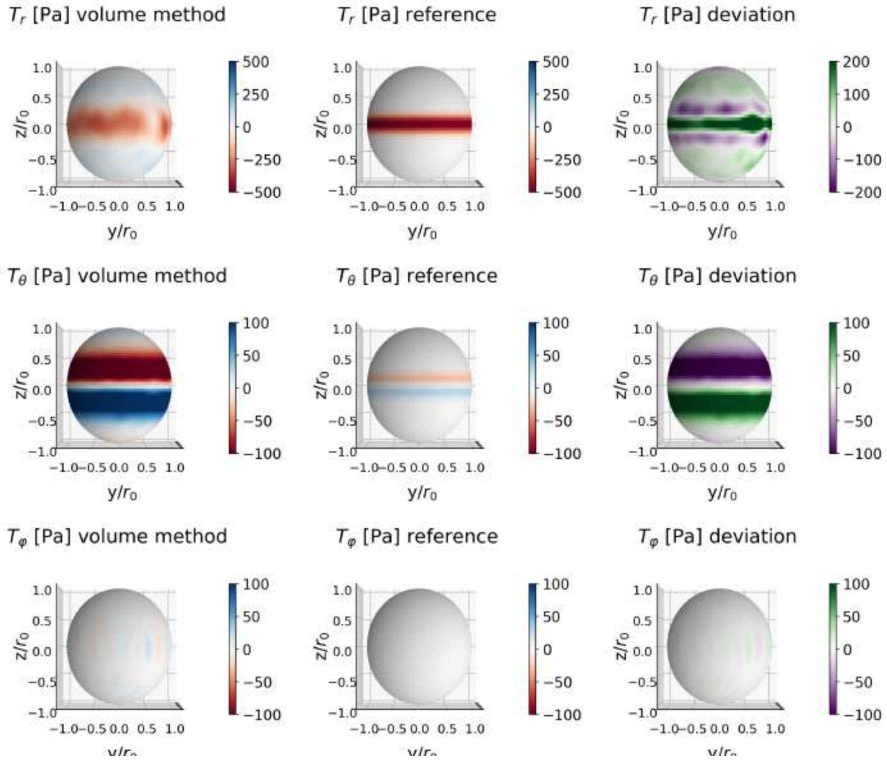

(a) Volume method.

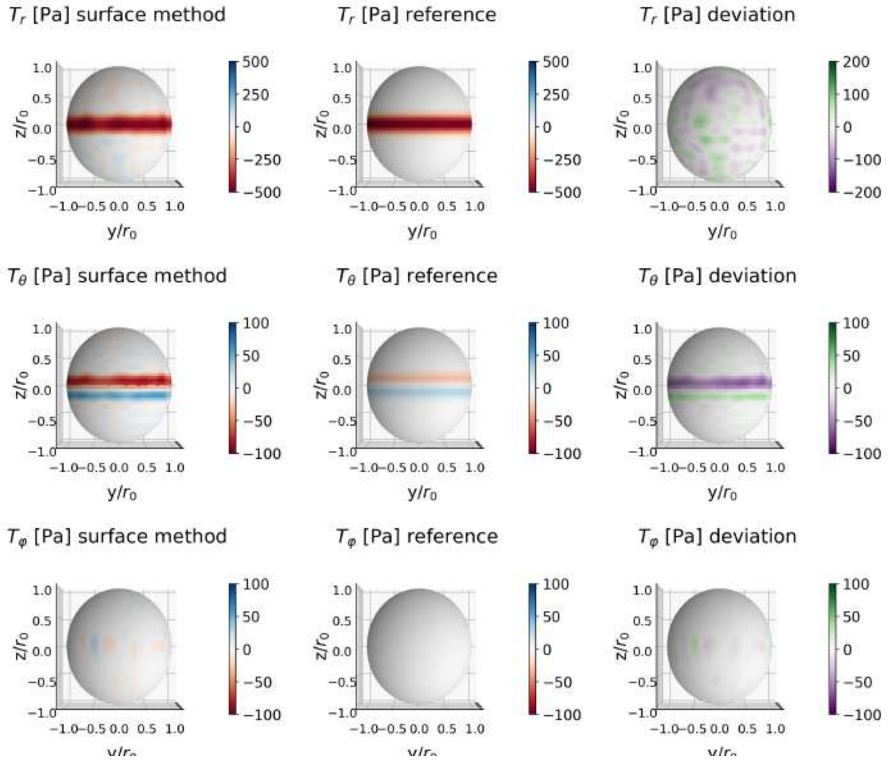

(b) Surface method.

Figure S12 Full simulation results of all three traction components for the ring scenario.

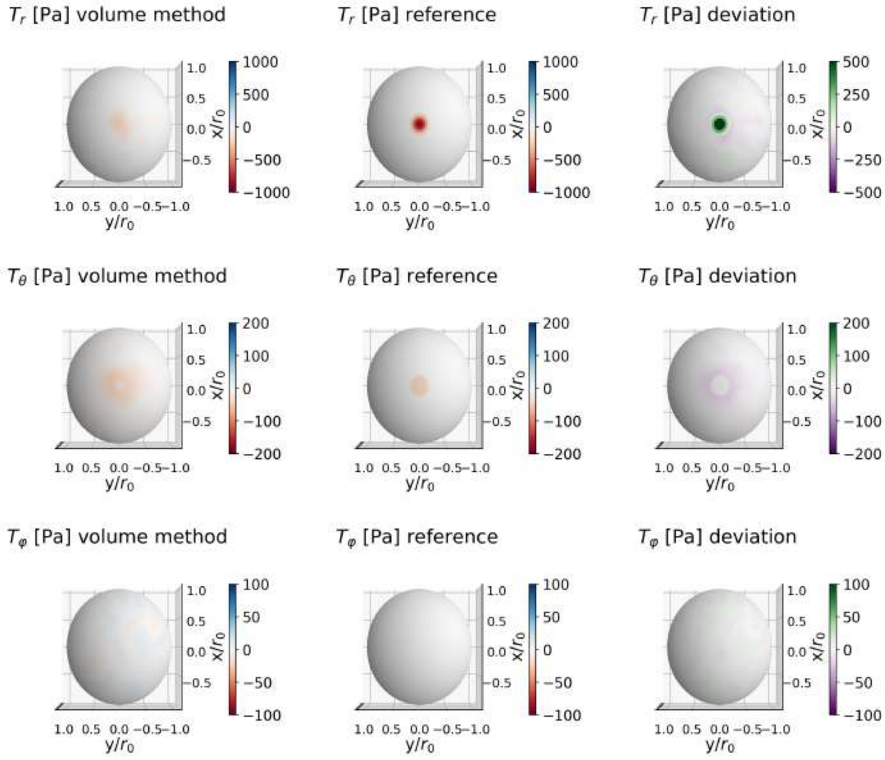

(a) Volume method.

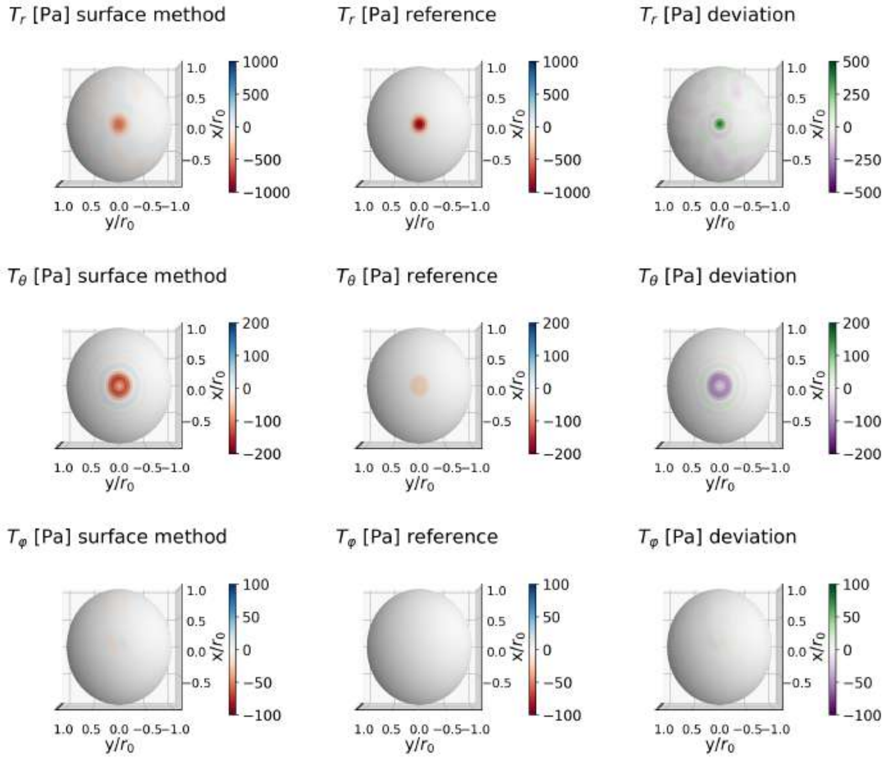

(b) Surface method.

Figure S13 Full simulation results of all three traction components for the indenter scenario.

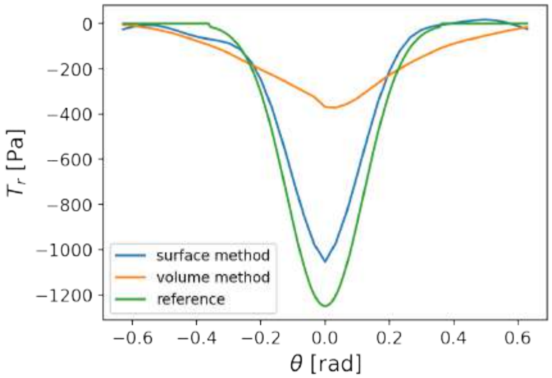

(a) $\theta_0 = 0.12$

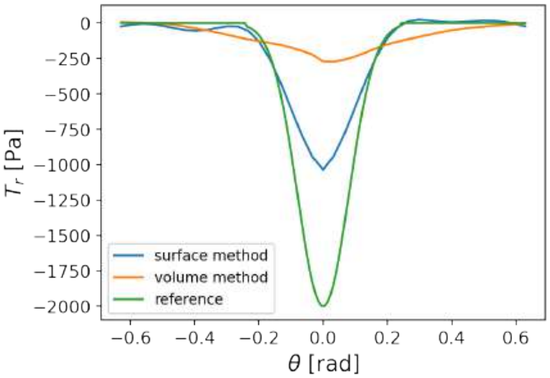

(b) $\theta_0 = 0.08$

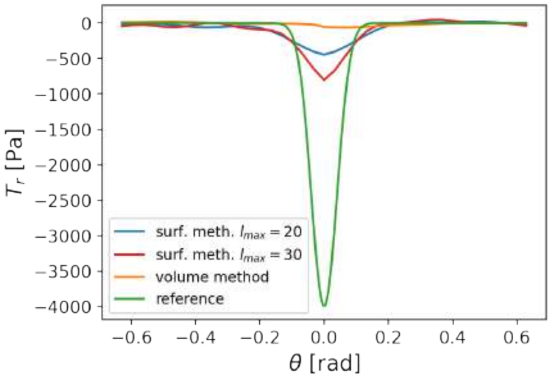

(c) $\theta_0 = 0.04$

Figure S14 Performance decrease of both methods for highly localized indentation. The accuracy in recovering the reference profile strongly decreases with a decrease of the indentation width θ_0. (a, b, c) Reference and recovered traction component T_r near the top pole of the sphere along y-axis for $x = 0$ (negative/positive θ corresponds to negative/positive y values). The width θ_0 of the Gaussian indenter is varied in (a, b, c) as stated. The magnitude is varied accordingly to obtain similar indentation depths in all cases ($\approx 20\%$). The results in Fig.2 correspond to the parameters used in (b).

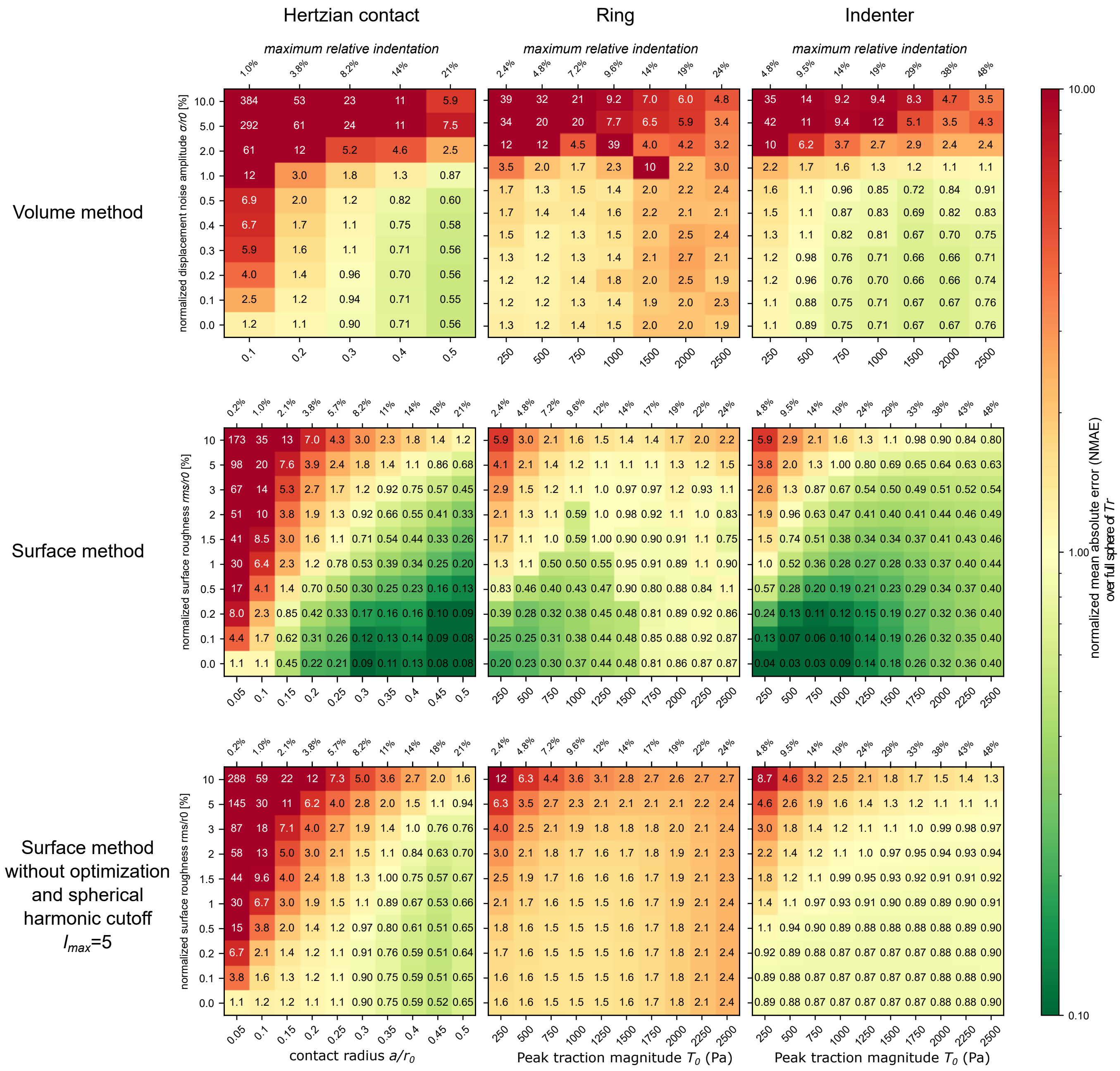

Figure S15 Full heatmap grids for method error comparison for full sphere evaluation of the NMAE of T_r.

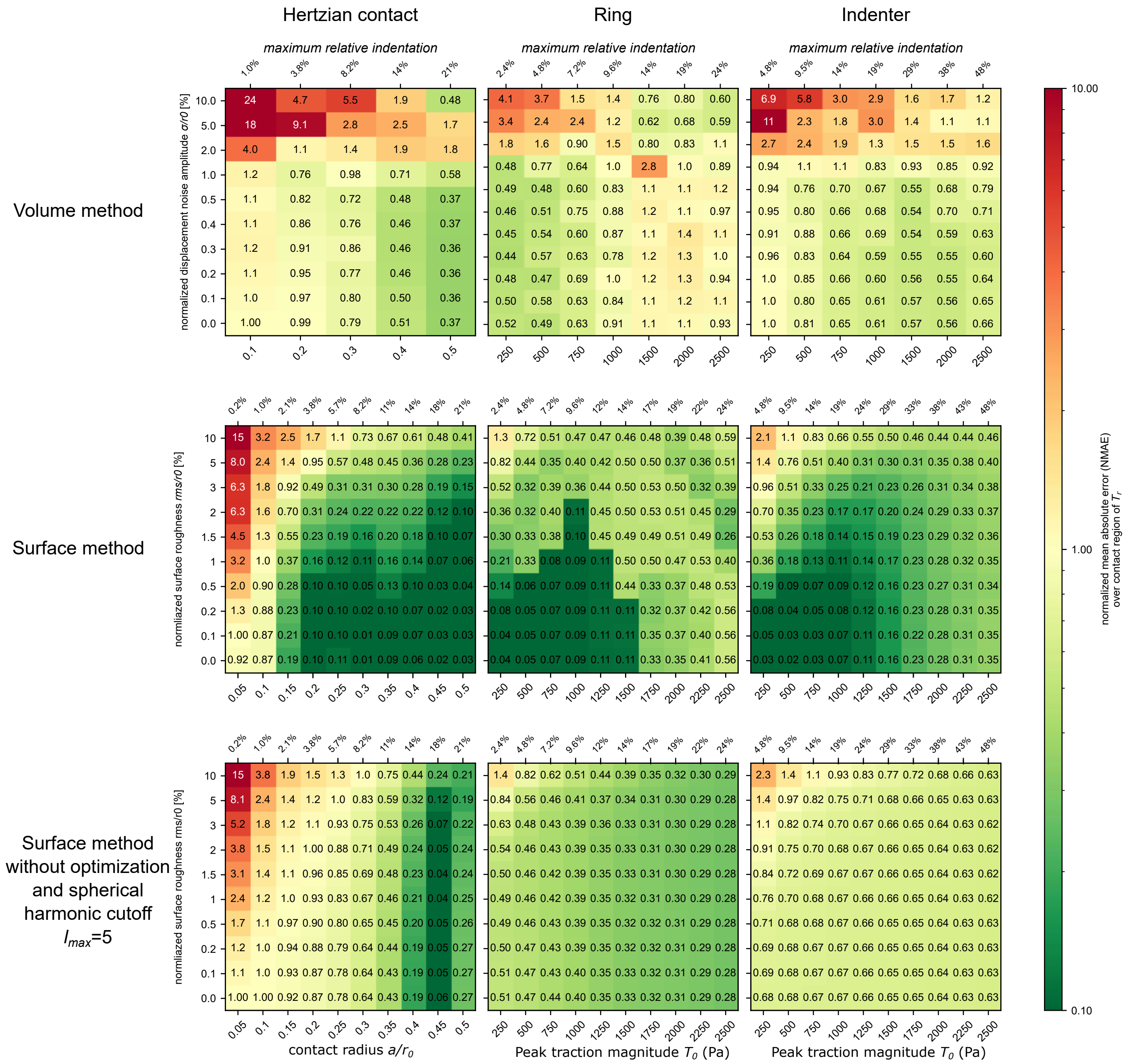

Figure S16 Heatmap grids for error evaluation restricted to dominant region. In Fig.3, the NMAE was evaluated across the whole sphere. Here we evaluate the NMAE in a region close to the region of maximum force application only. The same formula from Eq.(15) is used, however here we only sum over a smaller region Ω_0. For the Hertzian contact and indenter profile, we choose a region near the poles close to the z-axis (i.e. $\theta \in \Omega_0 = [0°, 9°] \cup [171°, 180°]$); for the ring profile close to the equator ($\Omega_0 = [81°, 99°]$).

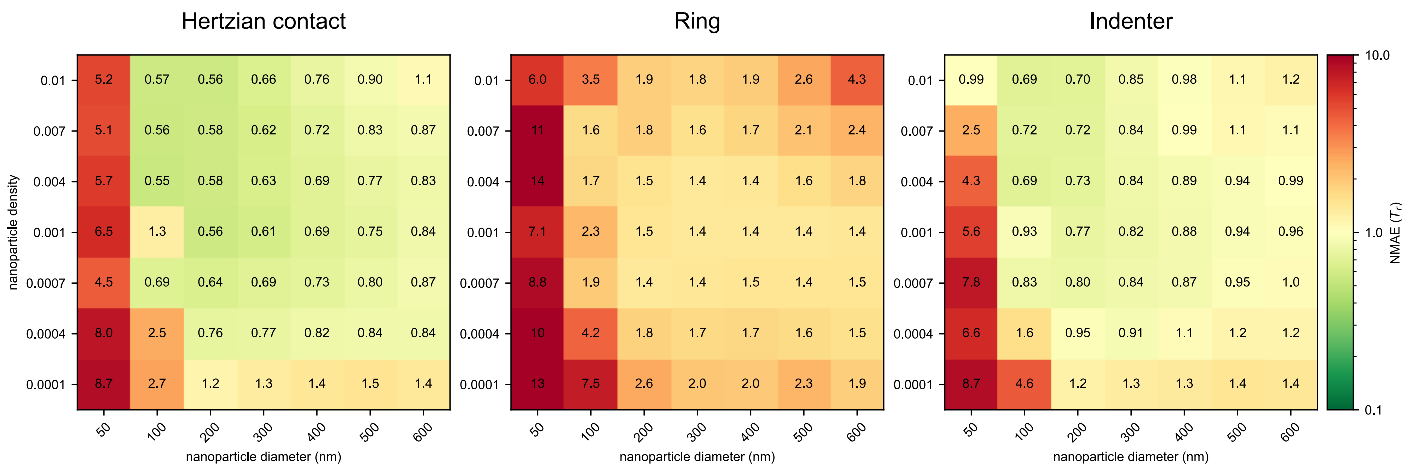

(a) Reconstruction error as a function of nanoparticle density and diameter. Heatmaps show the full sphere NMAE of the radial traction component T_r recovered by the volume method for the Hertzian contact, ring, and indenter scenarios as a function of nanoparticle diameter and density (given in nanoparticles/voxel). Each panel uses the same color scale as Fig. 3 to enable direct comparison. Optimal parameter regions differ between loading scenarios, and very small nanoparticle diameters (50 nm) result in increased errors across all cases, consistent with the nanoparticle size approaching the resolution limit.

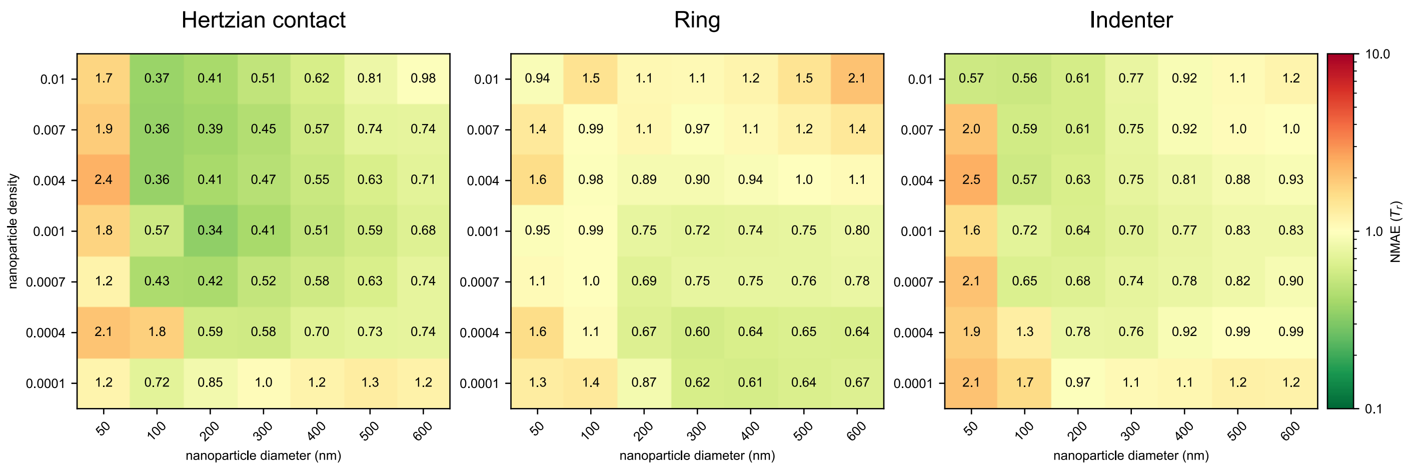

(b) Reconstruction error in contact region as a function of nanoparticle density and diameter. Here, the NMAE of T_r was evaluated only in a contact region around the force application, leading to reduced error metrics that still show similar limitations as the full sphere evaluations. However, the optimum for the ring seems to be quite different, shifting to higher nanoparticle diameters and lower densities.

Figure S17 Effect of nanoparticle properties on volume method accuracy. Fig. 3 shows that, under representative parameter settings, the volume method exhibits larger reconstruction errors than the surface method. Because the volume approach relies on displacement tracking of embedded fluorescent nanoparticles, its accuracy may depend on density and diameter of these fiducial markers. We therefore systematically varied these experimental parameters to assess whether improved marker-based displacement resolution reduces reconstruction errors, with full results for each configuration shown above. Importantly, we find that although suitable parameter choices reduce the error magnitude in specific regimes, even at the respective optimal conditions, the minimum reconstruction error of the volume method remains above that achieved by the surface method.

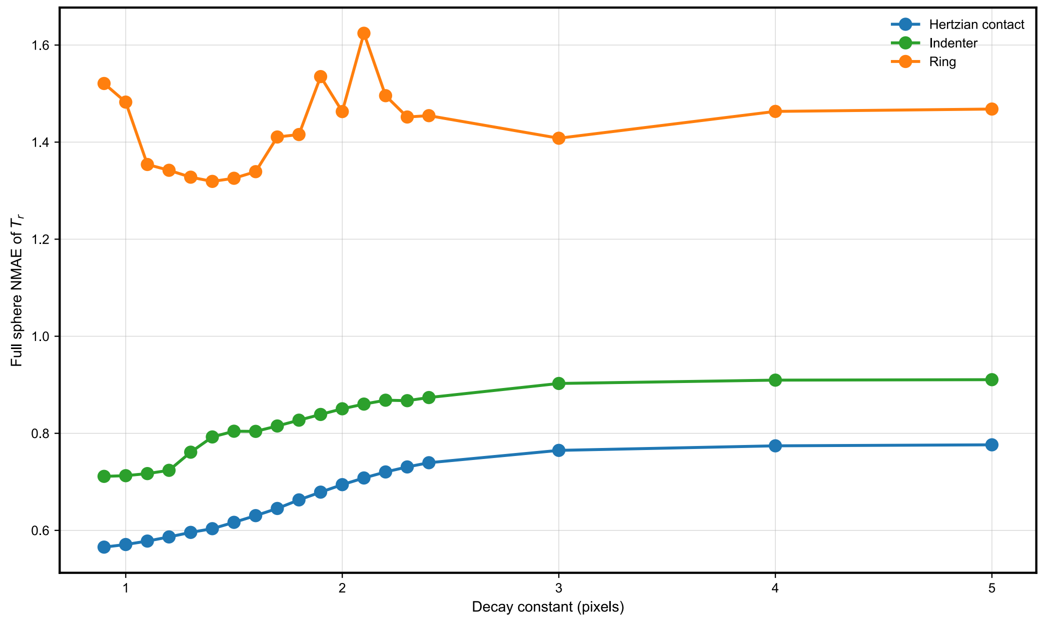

(a) NMAE full sphere.

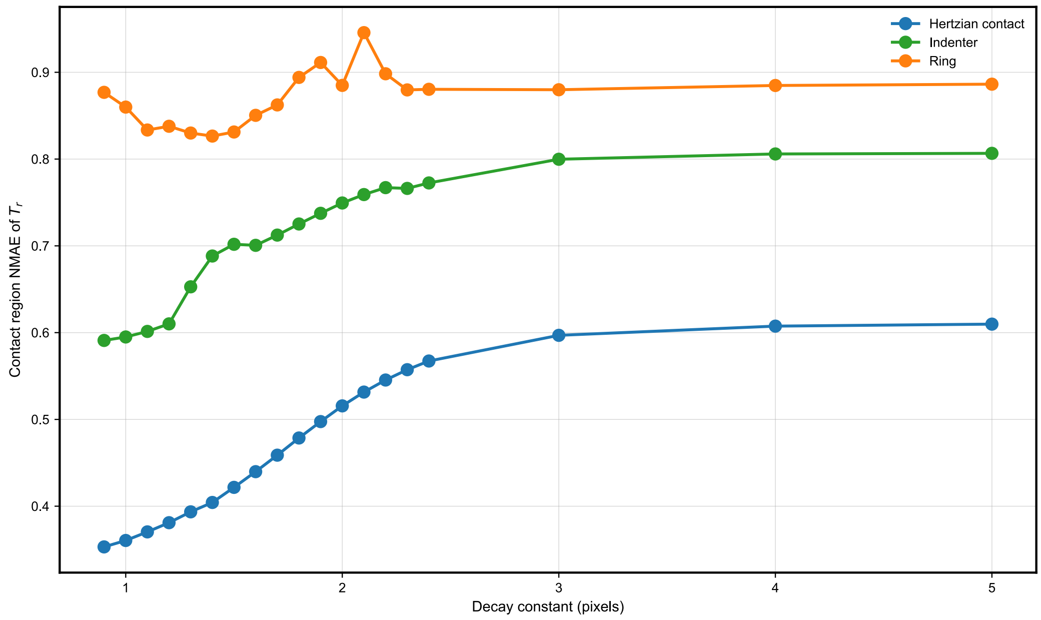

(b) NMAE contact region.

Figure S18 Simulation results for imaging artifact sensitivity via PSF decay constant sweep, mimicking different blurring levels. The decay constant γ_d controls the width of the PSF applied to the simulated nanoparticles in volume method image generation, with larger decay values representing heavier optical blur. The PSF as a function of the distance r from the center of the nanoparticle is defined as

$$\mathrm{PSF}(r) = I_0 \cdot \exp\left(-\frac{1}{2}\left(\frac{r}{\gamma_d}\right)^4\right), \tag{20}$$

where I_0 denotes the peak intensity in the center of the microparticle. Both r and γ_d are given in pixels. γ_d ranges from 0.9 (minimal blur, sharp PSF) to 5 (heavy blur, degraded optical conditions). Standard analyses in this work used a value of 1. NMAE of T_r is shown as a function of PSF decay constant, evaluated either over the full sphere (a) or only within the contact region (b), the latter isolating the regions where traction is applied and therefore revealing sensitivity to local force features. The analysis quantifies errors in reconstructing the prescribed traction profiles under increasing imaging blur and reveals scenario-dependent robustness. Localized contact geometries, including Hertzian contact and indenter cases, show substantial error degradation with increasing PSF blur, with an approximately 30% error increase from decay 0.9 to 5, reflecting the requirement for sharp image definition to accurately resolve localized contact boundaries for particle correlation. In contrast, the distributed stress gradient for the ring indenter remains robust to PSF blur, with only approximately 5% error variation across the decay range. These results suggest that contact geometry type fundamentally determines sensitivity to imaging degradation.

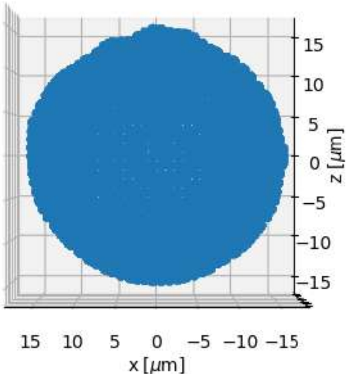

(a) Undeformed reference surface mesh of the DNA-HMP.

Condition	RMS sphericity deviation		
	overall	bottom	top
Reference (undeformed)	3.3%	2.6 %	3.9 %
Deformed (maximum load)	5.5%	5.5%	5.5%
7 min after loading	4.3%	4.2%	4.3%
25 min after loading	3.8%	3.8%	3.8%

(b) RMS deviation from a best-fit sphere.

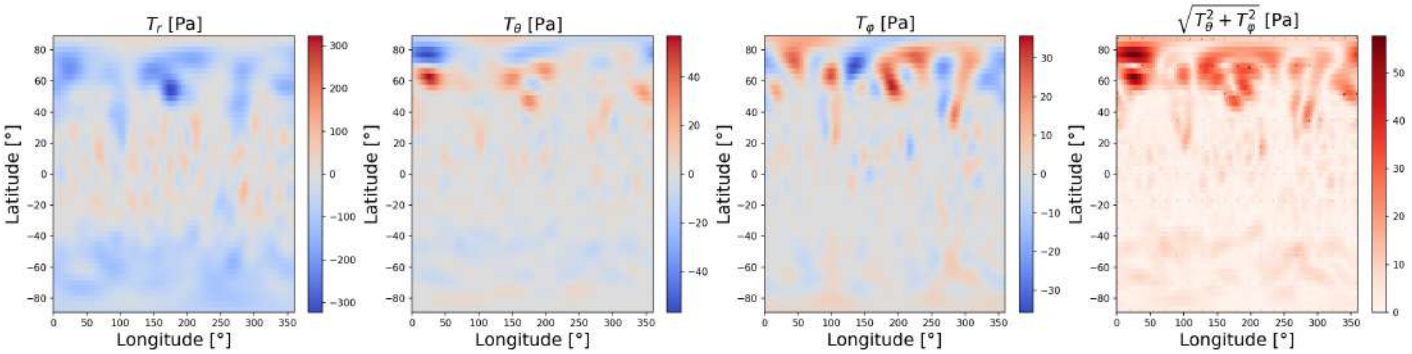

(c) Surface method traction reconstruction of the undeformed reference state.

Figure S19 Quantification of sphericity deviation and residual tractions of DNA-HMP reference configurations. (a) Surface point cloud of the undeformed reference configuration of the DNA-HMP analyzed in the main text. The reference configuration remains overall spherical, supporting the undeformed spherical reference assumption. (b) RMS sphericity deviations from a best-fit sphere for different experimental conditions. After deformation, the RMS values return close to initial RMS values, indicating that the sphere returns to a spherical shape. Corresponds to Fig.Fig.S22(a-d). RMS sphericity deviation is defined as the root-mean-square of the radial residuals normalized by the fitted sphere radius, providing a size-independent measure of shape distortion. (c) Surface method traction reconstruction of the undeformed reference configuration, showing the three traction components T_r, T_θ, and T_φ, together with the shear traction magnitude. The traction magnitudes observed are low compared to that of the deformed state analyzed in the main text, particularly on the bottom hemisphere where the surface is more spherical and imaging quality is higher. Residual tractions at the upper hemisphere could arise from initial geometric asymmetry, imaging artifacts, or minor forces exerted during well positioning prior to full loading. Together, these analyses support the validity of the undeformed spherical reference assumption used for DNA-HMP traction reconstruction.

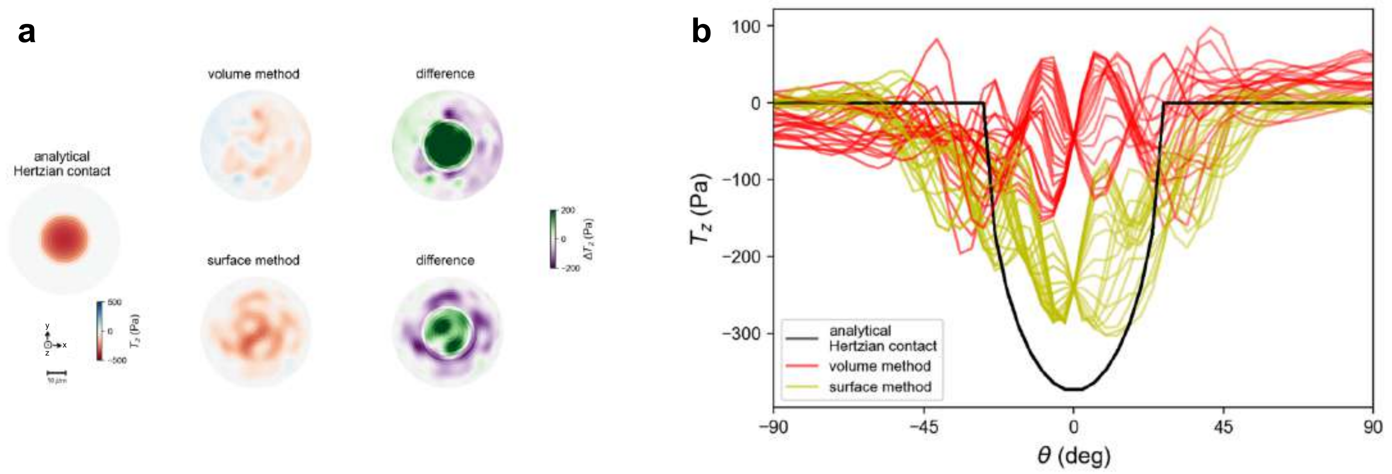

Figure S20 Analogous to Fig.5 (without the experimental setup in subfigures (a-d)), shown here are the reconstructed traction profiles of the top side of the same microparticle together with the same reference Hertzian contact ($a = 0.45$). (a) Analytical reference (left) and volume and surface method reconstructions (center) of T_z, together with the deviation from the reference (right). (b) Superimposed cross-sections. The resulting tractions do not reflect the Hertzian contact as well as the reconstructed bottom side profile in Fig.5(e,f), with the surface method capturing better correspondence. However, this might not be a shortcoming of the methods themselves: Due to our experimental setup, the deformation pattern at the top side where the glass slide was pushing down, as well as the image quality, was less clear, because the glass slide could move (while the bottom is fixed). Signal quality and resolution therefore were decreased. This is visualized (particularly for the surface method) in S21, which might also point at increased deformation at the bottom. The cross-sections still show that the compression can be captured by the surface method, and since the NMAE was evaluated for both methods over both contact regions (top and bottom side), reconstruction quality at the bottom is better than reflected by these numbers, implying higher quality traction profile reconstructions are possible with improved experimental setups.

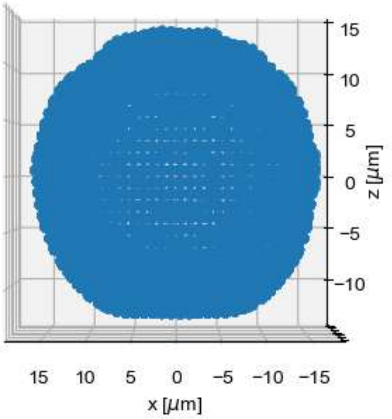

Figure S21 Set of points describing the microparticle surface of the DNA-HMP analyzed in Fig.5 and S20, obtained from using GeoV[47] (after ImageJ[46] preprocessing) on the microscope data of the Cy3-label in the DNA network. Noticeably, signal quality at the top seems decreased, and the deformation is less clearly visible (possibly also because the force application had a different contact radius on this side). Reasons for quality decline at the top might be that the glass slide is not fixed like the bottom, and optical effects leading to lower signal on top. While the set of points itself is only used in the surface method, these general experimental shortcomings could explain the worse top side reconstructions by both methods shown in Fig.S20, Fig.S23(b), and Fig.S24(b, d).

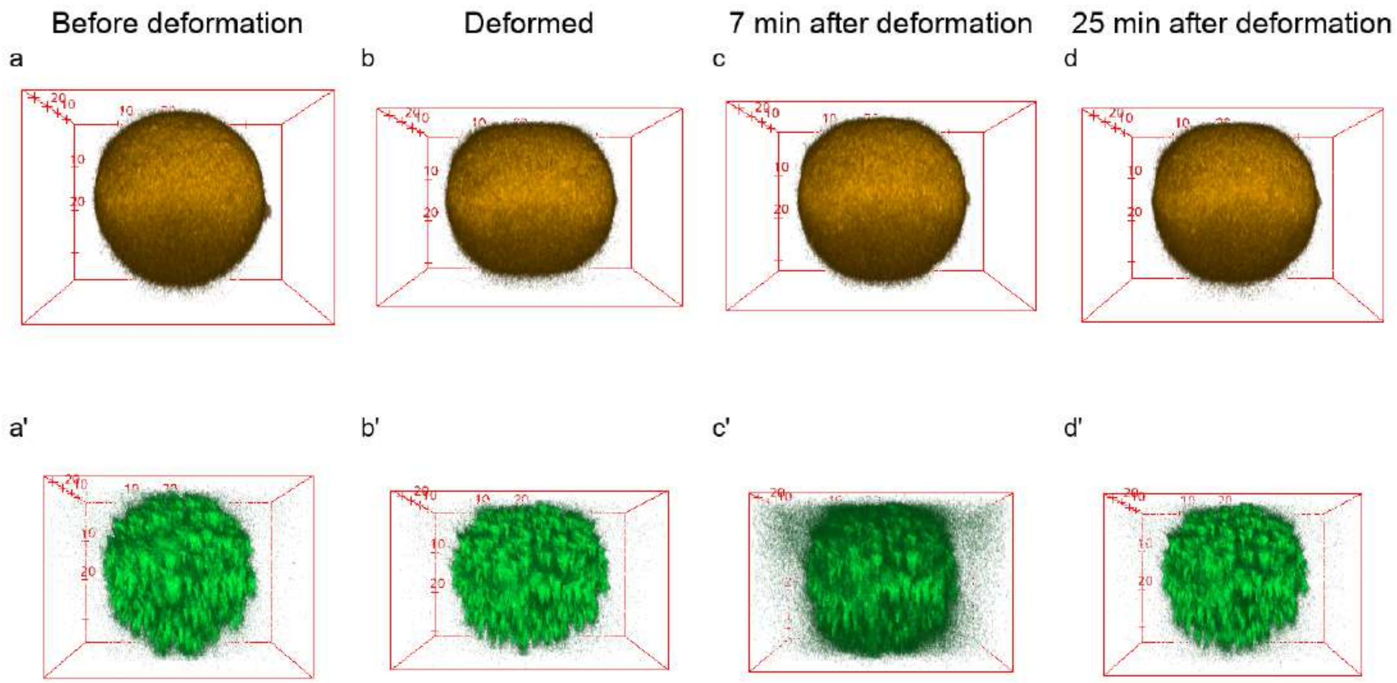

Figure S22 Microscope images of the DNA-HMP analyzed in the main text (Fig. 5), visualized using 3D Viewer in ImageJ.[46] Note the z-axis is pointing downwards here. First row (a, b, c, d) shows the Cy3 labels of the DNA network, used for the surface method reconstruction. Second row (a', b', c', d') shows the nanoparticles tracked in the volume method. From left to right, the columns represent the images taken before the deformation from the top by a weight, the deformed state, and two relaxation images captured after the weight was lifted (7 min and 25 min after). The volume method for the main text analysis used the relaxation of 25 min as reference image.

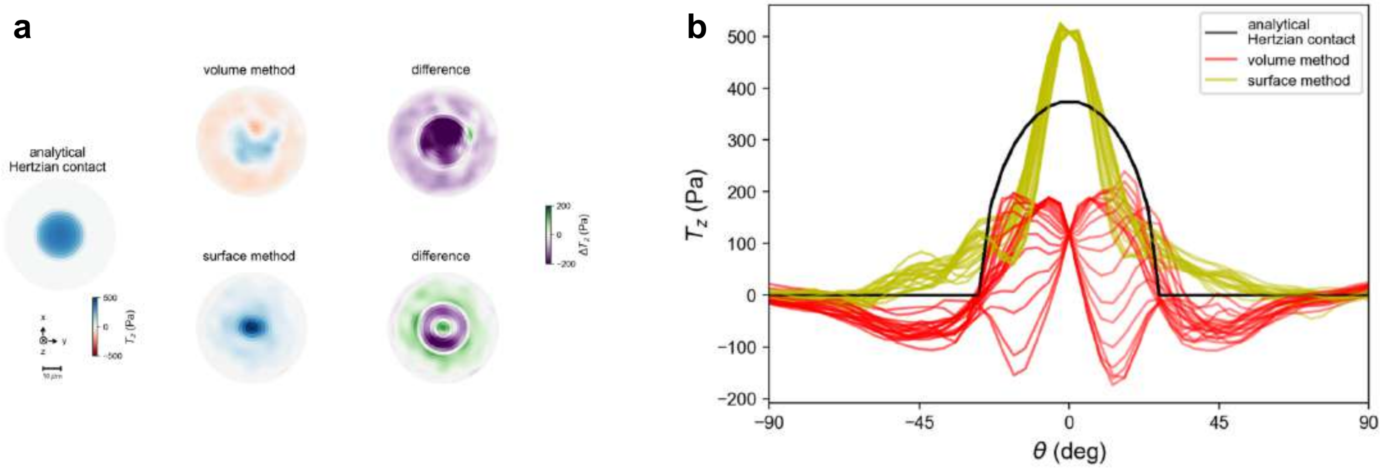

(a) Analogous to Fig.5(e,f), shown here is the bottom side traction reconstruction for another DNA-HMP. (a) Analytical reference (left, $a = 0.45$ for the Hertzian contact) and volume and surface method reconstructions (center) of T_z, together with the deviation from the reference (right). (b) Superimposed cross-sections. It seems the surface method overall better captured the compression than the volume method, but overestimated the traction magnitude in the center. The volume method underestimated the traction magnitudes, and the area of force application is less clearly resolved. Both methods captured an indentation, but the volume method also captured some shearing, which could be explained by slight microparticle rotation in the setup (which the reference-free surface method would not be able to detect).

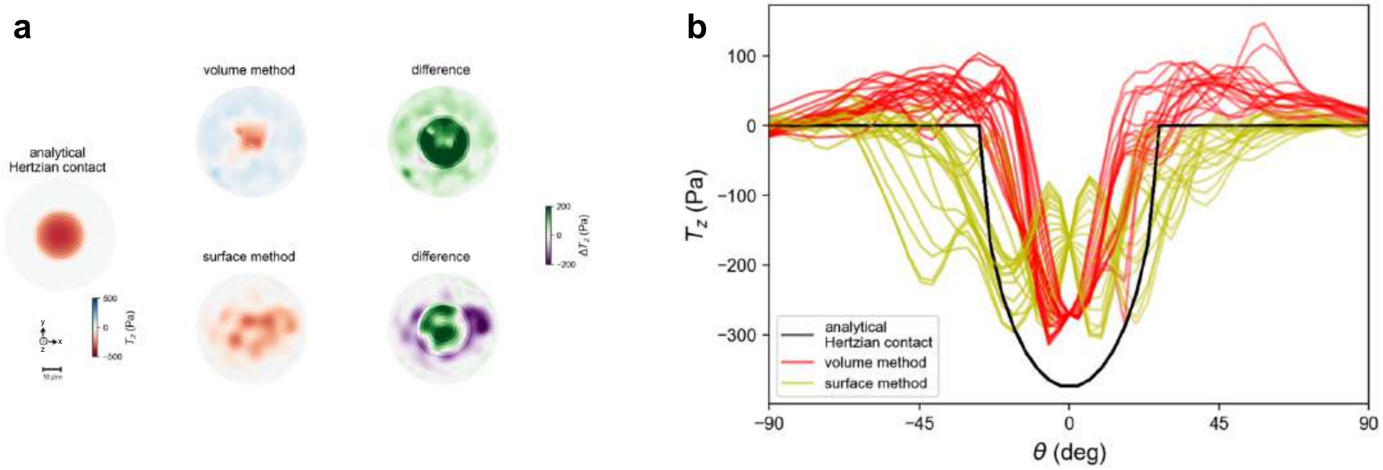

(b) Analogous analysis to Fig.S23a, but for the top side. (a) Analytical reference (left) and volume and surface method reconstructions (center) of T_z, together with the deviation from the reference (right). (b) Superimposed cross-sections. Both methods captured an indentation from this side, with the volume method having a slightly delocalized and underestimated profile, and the surface method (like in Fig.S20) again seeming less clear than on the bottom side, possibly due to the experimental setup (see also Fig.S21).

Figure S23 Traction reconstruction results for both surface and volume method for another DNA-HMP.

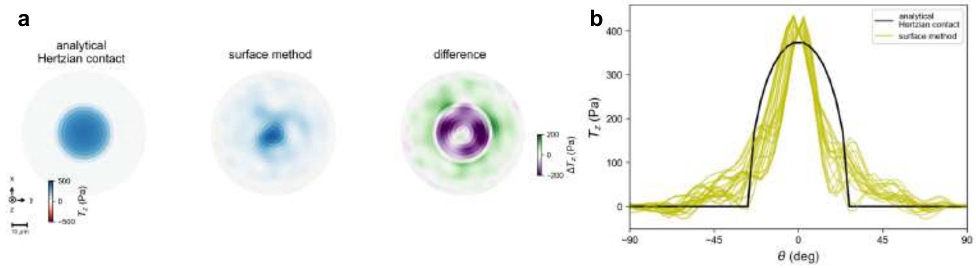

(a) Surface method results for yet another DNA-HMP, bottom view. (a) Analytical reference (left) and reconstruction (center) of T_z, together with the deviation from the reference (right). (b) Superimposed cross-sections. The compression is reconstructed, but underestimated outside of the center when comparing to the Hertzian contact profile with contact radius $a = 0.45$.

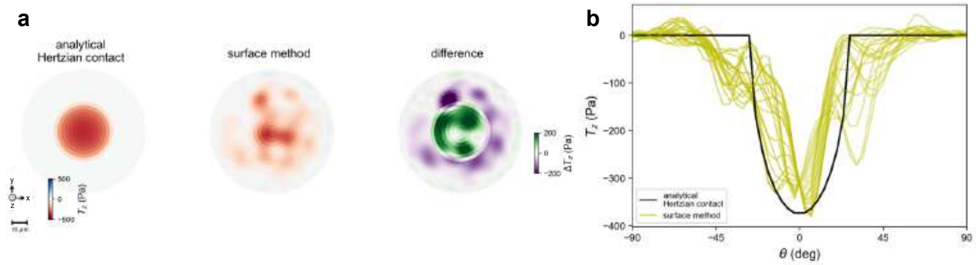

(b) Surface method results for the same DNA-HMP as in Fig. S24a, top view. (a) Analytical reference (left) and reconstruction (center) of T_z, together with the deviation from the reference (right). (b) Superimposed cross-sections. Similar trends as on the bottom can be observed for this perspective, but the center force application seems less localized.

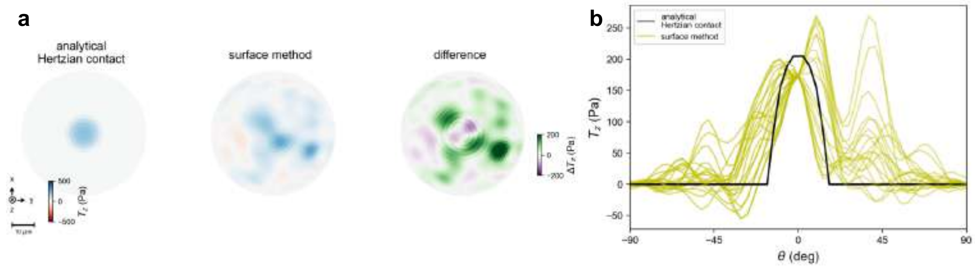

(c) Surface method results for a different DNA-HMP, bottom view. (a) Analytical reference (left, here using $a = 0.25$ for the Hertzian contact) and reconstruction (center) of T_z, together with the deviation from the reference (right). (b) Superimposed cross-sections. With the lowered contact radius, the reconstruction seems aligned to the Hertzian contact in the center, but away from the center, delocalized forces are reconstructed as well.

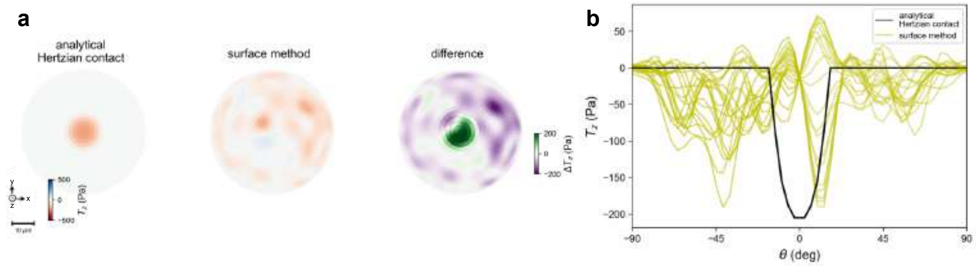

(d) Surface method results for the same DNA-HMP as in Fig. S24c, top view. (a) Analytical reference (left, here using $a = 0.25$ for the Hertzian contact) and reconstruction (center) of T_z, together with the deviation from the reference (right). (b) Superimposed cross-sections. The reconstructed applied force seems underestimated and off-center, which might be explained by asymmetric force application as well as the issues already outlined.

Figure S24 Surface method results for two more DNA-HMPs.

volume method

parameter	value
microparticle properties:	
radius r_0	12 μm
elastic modulus E	1500 Pa
Poisson's ratio ν	0.4
resolution	120 nm(x,y); 240 nm (z)
nanoparticle density ρ_n	0.002 nanoparticles/voxel
nanoparticle radius r_n	100 nm
cutoff $n_{\max}$	50
displacement noise σ_{disp}	0.17 px
padding factor p	1.28
resulting picture size	256x256x128†
output spacing FIDVC	4
gradient method	optimal-5
convergence criteria	default
evaluation radius r_e	0.8 r_0

surface method

parameter	value
microparticle properties:	
radius r_0	12 μm
elastic modulus E	1500 Pa
Poisson's ratio ν	0.4
cutoff $n_{\max}$	50
GLQ mesh order $l^*_{\max}$	50
deformed surface simulation parameters:	
number of surface (Fibonacci) points N	2000
roughness amplitude σ_r (relative to r_0)	0.02
power-law exponent β	3
minimum SH degree $l_{\min}$	2
minimization parameters:	
functional prefactors $\{\alpha, \beta, \gamma\}$	$\{1, 1, 0.3\}$‡
# iterations/period	10
# periods n_p	15
SH cutoff $l_{\max}$	20

Table S1 Standard simulation and analysis parameters for volume and surface method, used in this work if not stated otherwise. The lower resolution in the z-direction for the volume method resembles the lower resolution typically obtained by z-stack scanning in experiments.

† This picture size is very suitable for the FIDVC, as the sizes in all directions are powers of 2: Due to the window halving steps, other resolutions can cause asymmetric outputs of the FIDVC, which need to be accounted for in post-processing, unnecessarily complicating the evaluation procedure. The padding factor p, describing how much space we add around the sphere was chosen to obtain this resolution ($p = 1$ corresponds to the width of the picture being equal to the diameter of the sphere $2r_0$). This should also be taken into consideration by experimentalists when using this algorithm. The picture dimensions do not necessarily need to be an exact power of 2, it could also be a multiple of powers of 2, depending on the desired resolution. Choosing it to be a multiple of 32 is usually sufficient.

‡ These parameters were used in the original publication and shown to provide reasonable results in extensive numerical analyses.[29] Technically, they also resolve unit discrepancies between the energy functional terms.

Name	DNA sequence 5' - 3'
A-1	GACCAACACCAGTGAGGACGGAAGTTTGTCGTAGCATCGCACC
A-1-Chol	GACCAACACCAGTGAGGACGGAAGTTTGTCGTAGCATCGCACCTTT-Chol
A-2	GACCAACACCAACCACGCCTGTCCATTACTTCCGTCCTCACTG
A-3	GACCAACACGGTGCGATGCTACGACTTTGGACAGGCGTGGTTG
B-1	CAGTGAGGACGGAAGTTTGTCGTAGCATCGCACCCGACAGGAA
B-1-Cy3	Cy3-CAGTGAGGACGGAAGTTTGTCGTAGCATCGCACCCGACAGGAA
B-2	CAACCACGCCTGTCCATTACTTCCGTCCTCACTGCGACAGGAA
B-3	GGTGCGATGCTACGACTTTGGACAGGCGTGGTTGCGACAGGAA
Linker	GTGTTGGTCTTCCTGTCG

Table S2 DNA sequences used for the formation of the DNA-HMPs. The fluorescent label cyanine 3 is abbreviated as Cy3. Cholesterol is abbreviated as Chol.

Note S1: SH decomposition of displacement and traction

The decomposition described in this step is based on the work of Wang et al. in 2019.[36]. To evaluate the functional quickly, it is necessary to obtain a fast way to convert from $\vec{u}$ to $\vec{T}$, as the terms in the functional have an explicit dependence on $\vec{T}$. The idea here is to choose a basis for the displacement function space where each basis vector $\vec{u}^{(K)}$ corresponds to a basis vector $\vec{T}^{(K)}$, i.e. that if a displacement field is given as a superposition

$$\vec{u} = \sum_K a_K \vec{u}^{(K)}, \tag{21}$$

the corresponding traction is given by

$$\vec{T} = \sum_K a_K \vec{T}^{(K)}. \tag{22}$$

Such a basis can be constructed by expressing the quantities and thus also the basis vectors in terms of spherical harmonics (SH), as explained in the following: We start with the Papkovich-Neuber ansatz (see Eq.(9)). Using a potential ansatz, the general harmonic solutions are then given by

$$\psi_{general}(r,\theta,\varphi) = \sum_{l=0}^{\infty} \sum_{m=-l}^{+l} \left(C^R_{lm} r^l + \frac{C^L_{lm}}{r^{l+1}} \right) Y_l^m(\theta,\varphi). \tag{23}$$

The part of the radial solutions with negative exponent in r lead to solutions for $\vec{u}$ diverging at the origin, so we set $C^L_{lm} = 0$. As we want to get a set of basis vectors, we use that the spherical harmonics Y_l^m provide an orthonormal basis for functions of the angles (θ,φ) projecting onto $\mathbb{R}$ or $\mathbb{C}$ and use them as a basis. As ψ_k (and later u_k and T_k) with $k \in [x,y,z]$ contain three components each, we need a full set of SH for each component k separately to cover the full solution space. Taking this into account, we choose a set of 3 numbers $K = (k,l,m)$ to denote our basis vectors as

$$\vec{\psi}^{(K)} = \vec{\psi}^{(k,l,m)}(r,\theta,\varphi) = \vec{e}_k r^l Y_l^m(\theta,\varphi). \tag{24}$$

Switching to index notation, we now plug the potential basis vectors into Eq.(9) and obtain*

$$[\vec{u}^{(klm)}]_j = u_j^{(klm)} = -4(1-\nu)\delta_{jk} r^l Y_l^m + \partial_j (r^{l+1} [\vec{Y}_l^m]_k). \tag{25}$$

Here we have set $\psi_0 = 0$, as we can recreate any ψ_0 with a suitable choice of coefficients in the $\vec{r} \cdot \vec{\psi}$-part in the same bracket.[36] We also introduced the first vector spherical harmonics (VSH1) $\vec{Y}_l^m = \vec{e}_r Y_l^m$. The single components k of the VSH1 can be decomposed into a superposition of scalar SH with coefficients Q:[36]

$$[\vec{Y}_l^m]_k = \sum_{l'} \sum_{m'} Q_{klm}^{l'm'} Y_{l'}^{m'} \tag{26}$$

Plugging this into Eq.(25) and executing the derivative, we get

$$u_j^{(klm)} = -4(1-\nu)\delta_{jk} r^l Y_l^m + r^l \sum_{l'} \sum_{m'} Q_{klm}^{l'm'} (l [\vec{Y}_{l'}^{m'}]_j + [\vec{\Psi}_{l'}^{m'}]), \tag{27}$$

introducing the second vector spherical harmonics (VSH2) $\vec{\Psi}_l^m = r\vec{\nabla} Y_l^m$. Similarly to the VSH1, their components can be decomposed into SH using a second set of coefficients P:

$$[\vec{\Psi}_l^m]_k = \sum_{l'} \sum_{m'} P_{klm}^{l'm'} Y_{l'}^{m'} \tag{28}$$

* We want to point out the difference between the indices j and k at this point to avoid confusion: k is part of the indication of the basis vectors (next to l and m). $j \in [x,y,z]$ specifies the components of that basis vector. For the initial definition of the basis vectors in Eq.(24) with $\vec{\psi}^{(K)}$, there is no use in distinguishing the two: As they are defined using $\vec{e}_k$, only the component $j = k$ is non-vanishing. However, this is not necessarily true for the other basis vectors like $\vec{u}^{(K)}$, containing derivatives of $\vec{\psi}^{(K)}$ as the ∂_j in Eq.(25). Hence, in this and subsequent equations, the additional index j is necessary.

Replacing the VHS2 in Eq.(27) with their SH decomposition, and doing so as well for the VSH1 once more yields:

$$u_j^{(klm)}(r,\theta,\varphi) = r^l \underbrace{\left[-4(1-\nu)\delta_{jk}Y_l^m + \sum_{l',m'} Q_{klm}^{l'm'} \sum_{l'',m''} (lQ_{jl'm'}^{l''m''} + P_{jl'm'}^{l''m''})Y_{l''}^{m''}\right]}_{U_j^{(klm)}(\theta,\varphi)} \tag{29}$$

Here we factored out the polynomial in r. Identifying the r-independent part $U_j^{(klm)}(\theta,\varphi)$, we can now further decompose it. Explicitly factoring out the sum $\sum_{l'',m''}$ in the second term and considering the first term, we can separate the SH and define the resulting coefficients as $\hat{U}_{jl'm'}^{(K)}$:

$$U_j^{(K)} = U_j^{(klm)}(\theta,\varphi) = \sum_{l'=0}^{l'_{\max}} \sum_{m'=-l'}^{l'} \hat{U}_{jl'm'}^{(K)} Y_{l'}^{m'}(\theta,\varphi) \tag{30}$$

As a result, each basis vector is now fully described by a decomposition into spherical harmonics. Here we introduced a cutoff $l_{\max}$, which can be set to favor either computational speed (low $l_{\max}$) or accuracy (high $l_{\max}$).

For the next step, we use the spherical versions of the strain and stress tensor:[52]

$$\begin{aligned}
u_{rr} &= \frac{\partial u_r}{\partial r}, & 2u_{r\theta} &= \frac{1}{r}\frac{\partial u_r}{\partial \theta} + \frac{\partial u_\theta}{\partial r} - \frac{u_\theta}{r},\\
u_{\theta\theta} &= \frac{1}{r}\left(\frac{\partial u_\theta}{\partial \theta} + u_r\right), & 2u_{r\varphi} &= \frac{1}{r\sin\theta}\frac{\partial u_r}{\partial \varphi} + \frac{\partial u_\varphi}{\partial r} - \frac{u_\varphi}{r}\\
u_{\varphi\varphi} &= \frac{1}{r\sin\theta}\frac{\partial u_\varphi}{\partial \varphi} + \frac{u_r}{r} + \frac{\cot\theta u_\theta}{r}, & 2u_{\theta\varphi} &= \frac{1}{r\sin\theta}\frac{\partial u_\theta}{\partial \varphi} + \frac{1}{r}\frac{\partial u_\varphi}{\partial \theta} - \frac{\cot\theta u_\varphi}{r}
\end{aligned} \tag{31}$$

$$\begin{aligned}
\sigma_{rr} &= (\lambda+2\mu)u_{rr} + \lambda u_{\theta\theta} + \lambda u_{\varphi\varphi}, & \sigma_{r\theta} &= 2\mu u_{r\theta},\\
\sigma_{\theta\theta} &= \lambda u_{rr} + (\lambda+2\mu)u_{\theta\theta} + \lambda u_{\varphi\varphi}, & \sigma_{r\varphi} &= 2\mu u_{r\varphi},\\
\sigma_{\varphi\varphi} &= \lambda u_{rr} + \lambda u_{\theta\theta} + (\lambda+2\mu)u_{\varphi\varphi}, & \sigma_{\theta\varphi} &= 2\mu u_{\theta\varphi}.
\end{aligned} \tag{32}$$

Evaluating Eq.(4), which can also be rewritten in terms of spherical harmonics, we obtain a similar separation and decomposition for the traction

$$T_j^{(K)} = T_j^{(k,l,m)} = r_0^{l-1} F_j^{(K)}(\theta,\varphi) = \sum_{l'=0}^{l_{\max}} \sum_{m'=-l'}^{l'} \hat{F}_{jl'm'}^{(K)} Y_{l'}^{m'}(\theta,\varphi). \tag{33}$$

$F_j^{(K)}$ and $\hat{F}_{jl'm'}^{(K)}$ denote the r-independent part of the traction and its decomposition coefficients for the respective basis vectors.

The coefficients $\hat{U}_{jl'm'}^{(K)}$ and $\hat{F}_{jl'm'}^{(K)}$ are fixed for a given set of material parameters (E, ν, r_0), as the relations between the SH and VSH Q and P are also constant. Thus, they can easily be precomputed and used again in each iteration of the minimization.

Note S2: Vector representation of the elastic energy

The subsequent procedure was introduced in the github package SHElastic.[53] The general package has been developed for the publication describing the SH decomposition of the previous step.[36] Example 06 of the package is the specific implementation for the surface method,[29] including suitable vector representations and reformulations of f, which we will present in the following.

The definitions Eq.(30) and Eq.(33) allow us to easily represent the full displacement field as a vector $\vec{\hat{U}}_{\mathrm{SH}}$, whose entries are the coefficients of the corresponding spherical harmonic (running from Y_0^0 to $Y_{l_{\max}}^{l_{\max}}$ for the three displacement field components u_x, u_y, u_z successively).The vector representation also allows us to rewrite the functional in such a way that we can efficiently perform the minimization.

We also need a vector representation for the traction in position space to evaluate the E_{res} term easily. To do so, we choose a set of N points on the surface, characterized by their corresponding angles (φ_i, θ_i). We choose a vector $\vec{\hat{U}}_{\mathrm{mesh}}$, whose entries simply describe the displacement values at the respective points i (The first i components describe u_x at the respective point, components $N+i$ describe u_y, etc.).

As the set of points, we use a GLQ mesh (Gauss-Legendre quadrature mesh, characterized by order l^*, which we choose to be the same as the SH, i.e. $l^* = l_{\max}$). This mesh is used as it facilitates a fast conversion between the mesh and the SH coefficient representation of the displacement field: It can be implemented as a matrix multiplication with a precomputed complex conversion matrix $\mathbf{S}$ (performed using *SHTools*[54]):

$$\vec{\hat{U}}_{\mathrm{SH}} = \mathbf{D}\vec{A}_K = \mathbf{S}\vec{\hat{U}}_{\mathrm{mesh}} \tag{34}$$

This intermediate definition resembles Eq.(21) in vector representation, where the coefficients a_K are represented as one vector $\vec{A_K}$. The entries of the matrix $\mathbf{D}$ consist of the coefficients $\hat{U}^{(K)}_{jl'm'}$ to relate the contributions of the corresponding basis vector a_K to the SH modes. For the traction, we similarly find:

$$\vec{\hat{T}}_{\mathrm{SH}} = \mathbf{C}\vec{A}_K = \mathbf{S}\vec{\hat{T}}_{\mathrm{mesh}} \tag{35}$$

We can relate the traction and displacement via $\vec{A_K}$ in the two equations, leading to:

$$\vec{\hat{T}}_{\mathrm{SH}} = \mathbf{C}\mathbf{D}^{-1}\mathbf{S}\vec{\hat{U}}_{\mathrm{mesh}} \tag{36}$$

Plugging Eq.(35) into Eq.(36), we can directly relate $\vec{\hat{T}}_{\mathrm{mesh}}$ and $\vec{\hat{U}}_{\mathrm{mesh}}$:

$$\vec{\hat{T}}_{\mathrm{mesh}} = \underbrace{\mathbf{S}^{-1}\mathbf{C}\mathbf{D}^{-1}\mathbf{S}}_{\mathbf{L}:=}\vec{\hat{U}}_{\mathrm{mesh}} = \mathbf{L}\vec{\hat{U}}_{\mathrm{mesh}} \tag{37}$$

Note that the vectors with the hat $\vec{\hat{U}}$ describe the displacement field $\vec{u}(\theta,\varphi)$, but other than $\vec{u}$, they are not vector fields themselves, but a vector representation of the field. The same holds true for the respective traction vectors.

With these definitions, we can rewrite the three different parts of our functional f. Using the definitions above, we also reformulate them to depend explicitly depend on $\vec{\hat{U}}_{\mathrm{mesh}}$, as we need to perform derivatives with respect to it later.

1. For R, we introduce a matrix $\mathbf{P}$ that selects the points of the mesh on the traction-free region and also contains weighing factors to compensate for the higher density of points near the poles for the GLQ mesh, which consists of $N = (l+1)(2l+1)$ points, and obtain

$$R = \frac{1}{N}\vec{\hat{T}}^T_{\mathrm{mesh}}\mathbf{P}\vec{\hat{T}}_{\mathrm{mesh}} = \frac{1}{N}(\mathbf{L}\vec{\hat{U}}_{\mathrm{mesh}})^T\mathbf{P}\mathbf{L}\vec{\hat{U}}_{\mathrm{mesh}}. \tag{38}$$

2. For E_{el}, instead of integrating across the surface, in the SH representation we can evaluate the integral with the orthogonality relation of the SH: $\int_{\theta=0}^{\pi}\int_{\varphi=0}^{2\pi} Y_\ell^m Y_{\ell'}^{m'*} d\Omega = 4\pi\delta_{\ell\ell'}\delta_{mm'}$. In the vector representation, this leads to

$$E_{\mathrm{el}} = \frac{1}{2}\vec{\hat{U}}_{\mathrm{SH}} \cdot \vec{\hat{T}}_{\mathrm{SH}} = 2\pi\mathbf{S}\vec{\hat{U}}_{\mathrm{mesh}}\mathbf{C}\mathbf{D}^{-1}\mathbf{S}\vec{\hat{U}}_{\mathrm{mesh}}. \tag{39}$$

3. We can now also define the high-frequency penalty in a meaningful way. For this, we use the matrix $\mathbf{Q}$ that introduces a damping coefficient for higher order traction modes,[29] which correspond to high spatial frequency components.

$$E_{\mathrm{pen}} = |\mathbf{Q}\vec{\hat{T}}_{\mathrm{SH}}|^2 = (\mathbf{Q}\mathbf{C}\mathbf{D}^{-1}\mathbf{S}\vec{\hat{U}}_{\mathrm{mesh}})^H \cdot (\mathbf{Q}\mathbf{C}\mathbf{D}^{-1}\mathbf{S}\vec{\hat{U}}_{\mathrm{mesh}}) \tag{40}$$

$(\ldots)^H$ denotes the Hermitian transpose to ensure we get the absolute value, as $\mathbf{S}$ contains complex elements.

Note S3: Minimization

In order to start the iteration, we need an initial guess for the displacement field. The easiest choice incorporating the measured shape of the deformed sphere is to assume the displacement is only happening in radial direction. As we know the surface shape of the sphere, we can determine the radial change for each point on the surface: Using linear interpolation, we define a function r^d_{interp} that, after calculating the volumetric central point $\vec{x}_0$ of the deformed sphere, assigns a radius value for the whole measured surface, parameterized by θ and φ. The radial displacement is then simply given as

$$u_r(\theta,\varphi) = r^d_{\text{interp}}(\theta,\varphi) - r_0, \tag{41}$$

where r_0 denotes the reference radius of the undeformed sphere. However, obtaining the true reference radius is unfortunately impossible without a reference picture. In theory, the sphere could have had any possible radius before, it could even have been compressed strongly and there would be no way of recovering this information from the single picture. The easiest solution for this is to compute the volume of the sphere and use the radius of a sphere of the same volume, given via $r_0 = \sqrt[3]{\frac{3V}{4\pi}}$.

With all the earlier stated advantages that taking only one picture brings, this is a major disadvantage of this method compared to the volume method: We basically assume that the material is incompressible and lose all information about isotropic normal traction components (pressure) acting on the sphere. We also assume the reference shape to be a perfect sphere, introducing another error source as most produced microparticle will probably slightly deviate from this shape. For the volume method, this is accounted for with the reference picture as well, containing any deviations from the spherical shape.

As stated before, we will use a GLQ mesh as the set of points i to perform the minimization on, each point being characterized by its position (θ_i,φ_i). As the surface shape is fixed, this limits the ways in which we can update the displacement during the minimization. To ensure that this boundary condition is always obtained, we choose the angular surface shifts $[\vec{u}_{\text{surf}}]_i = (\Delta\theta_i,\Delta\varphi_i)$ of the points i as the degrees of freedom that are updated throughout the minimization process. The surface shifts denote the change in solid angle from the reference mesh point. In this way, the corresponding radius can be determined from the interpolation function via $r^d_{\text{interp}}(\theta_i+\Delta\theta_i,\varphi_i+\Delta\varphi_i)$ and the boundary condition is always fulfilled. For the initial guess of the displacement field being only in the radial direction, this corresponds to $(\Delta\theta_i,\Delta\varphi_i) = (0,0)$ for every point i.

To update the field, we need the full Jacobian of the functional f with regard to the vector including all surface shifts $\vec{u}_{\text{surf}}$:

$$\frac{\partial f}{\partial \vec{u}_{\text{surf}}} = \frac{\partial f}{\partial \vec{U}_{\text{mesh}}}\frac{\partial \vec{U}_{\text{mesh}}}{\partial \vec{u}_{\text{surf}}} = \left(\alpha\frac{\partial R}{\partial \vec{U}_{\text{mesh}}} + \beta\frac{\partial E_{\text{el}}}{\partial \vec{U}_{\text{mesh}}} + \gamma\frac{\partial E_{\text{pen}}}{\partial \vec{U}_{\text{mesh}}}\right)\frac{\partial \vec{U}_{\text{mesh}}}{\partial \vec{u}_{\text{surf}}} \tag{42}$$

The entries of the Jacobian indicate how the functional changes with a change of the single surface shifts, representing a high-dimensional derivative. The derivatives of the three terms (R, E_{el}, E_{pen}) are given explicitly in example 6 of the corresponding GitHub package of the surface method.[53] To evaluate the second term $\frac{\partial \vec{U}_{\text{mesh}}}{\partial \vec{u}_{\text{surf}}}$, we need to account for the change of the interpolation function r^d_{interp} with regard to the surface shifts $(\Delta\theta_i,\Delta\varphi_i)$, as this directly determines the change in u_r. We approximate the derivative by calculating the 3-point gradient in a small environment around the respective position ($\varepsilon = 10^{-5}$) for each point i:

$$\frac{\partial u_{r,i}}{\partial \Delta\theta_i} = \underbrace{\frac{\partial u_{r,i}}{\partial r^d_{\text{interp}}}}_{=1}\frac{\partial r^d_{\text{interp}}}{\partial(\Delta\theta_i)} \approx \frac{r^d_{\text{interp}}(\theta_i+\varepsilon,\varphi_i) - r^d_{\text{interp}}(\theta_i-\varepsilon,\varphi_i)}{2\varepsilon} \tag{43}$$

$$\frac{\partial u_{r,i}}{\partial \Delta\varphi_i} = \underbrace{\frac{\partial u_{r,i}}{\partial r^d_{\text{interp}}}}_{=1}\frac{\partial r^d_{\text{interp}}}{\partial(\Delta\varphi_i)} \approx \frac{r^d_{\text{interp}}(\theta_i,\varphi_i+\varepsilon) - r^d_{\text{interp}}(\theta_i,\varphi_i-\varepsilon)}{2\varepsilon} \tag{44}$$

As they are given in radians and we can assume them to be small, the tangential displacement components are linear in

the small angular shifts after scaling with radius; therefore the corresponding Jacobian entries are:

$$\frac{\partial u_{\theta,i}}{\partial \Delta\theta_i} \approx r^d_{\text{interp}}(\theta_i, \varphi_i) \tag{45}$$

$$\frac{\partial u_{\theta,i}}{\partial \Delta\varphi_i} = 0 \tag{46}$$

$$\frac{\partial u_{\varphi,i}}{\partial \Delta\theta_i} = 0 \tag{47}$$

$$\frac{\partial u_{\varphi,i}}{\partial \Delta\varphi_i} \approx r^d_{\text{interp}}(\theta_i, \varphi_i) \tag{48}$$

Transferring these to Cartesian coordinates yields the needed relation.

The minimization itself is performed using the conjugate-gradient method.[55] As specific implementation, we use the *minimize* function of the *scipy.optimize* package.[56] The surface shifts are updated until we reach the maximum number of periods n_p.

Note S4: Traction profile definitions

Sphere-wall contact (Hertzian contact profile)

The Hertzian contact profile describes the contact between an elastic sphere and an elastic half-space (characterized by (E_0, ν) and (E_{hs}, ν_{hs}) respectively). It can be used to model the contact between an elastic sphere and a wall and is often utilized in AFM setups to estimate the elastic modulus E for materials. For the limit of small strains, this problem was solved in 1882 by Heinrich Hertz.[57] It is equivalent to the contact between a rigid wall and an elastic sphere with an effective elasticity modulus E^*:

$$\frac{1}{E^*} = \frac{1-\nu^2}{E_0} + \frac{1-\nu_{hs}^2}{E_{hs}} \tag{49}$$

The resulting traction field on the sphere is originally parallel to the z-direction,[58] decomposing it via $T_r = T_z \cos(\theta)$ and $|T_\theta| = |T_z \sin(\theta)|$ yields:

$$T_r(\theta) = \begin{cases} -\frac{2E^*}{\pi r_0}\sqrt{a^2 - r_0^2 \sin(\theta)^2}|\cos(\theta)| & \text{for } r_0 \sin(\theta) \le a \\ 0 & \text{for } r_0 \sin(\theta) > a \end{cases} \tag{50}$$

$$T_\theta(\theta) = \begin{cases} \pm\frac{2E^*}{\pi r_0}\sqrt{a^2 - r_0^2 \sin(\theta)^2}\sin(\theta) & \text{for } r_0 \sin(\theta) \le a \text{ and } \theta \lessgtr \pi/2 \\ 0 & \text{for } r_0 \sin(\theta) > a \end{cases} \tag{51}$$

a describes the radius of the area where the sphere touches the wall, demonstrated in Fig.2. It is connected to the total applied force F via

$$a = \sqrt[3]{\frac{3Fr_0}{4E^*}}. \tag{52}$$

Gaussian indenter

One example of a more localized profile is that of an indenter pushing on the top and bottom of the sphere. We model this scenario by applying a localized Gaussian distributed traction profile of width θ_0, resembling the standard deviation. We parametrize the Gaussian directly via θ:

$$T_r(\theta) = T_0 \cdot \begin{cases} -\exp(-\theta^2/(2\theta_0^2))\cos(\theta) & \text{for } 0 \le \theta \le \theta_{cut} \\ 0 & \text{for } \theta_{cut} < \theta < \pi - \theta_{cut} \\ \exp(-(\pi-\theta)^2/(2\theta_0^2))\cos(\theta) & \text{for } \pi - \theta_{cut} \le \theta \le \pi \end{cases} \tag{53}$$

$$T_\theta(\theta) = T_0 \cdot \begin{cases} -\exp(-\theta^2/(2\theta_0^2))\sin(\theta) & \text{for } 0 \le \theta \le \theta_{cut} \\ 0 & \text{for } \theta_{cut} < \theta < \pi - \theta_{cut} \\ \exp(-(\pi-\theta)^2/(2\theta_0^2))\sin(\theta) & \text{for } \pi - \theta_{cut} \le \theta \le \pi \end{cases} \tag{54}$$

We introduced a cutoff angle θ_{cut}, which is generally 3 times the size of θ_0. In this way, we do not unnecessarily shrink the traction free-region for the surface method, but still get a smooth profile. θ_0 and θ_{cut} are usually small. Considering the small angle approximation, we can assume that θ_0 corresponds to a contact/cutoff radius in terms of r_0. For example, if $\theta_0 = 0.08$, the width of the Gaussian corresponds to 8% of the radius of the sphere ($\approx 1\mu$m for $r_0 = 12\mu$m in the simulations).

Gaussian ring

Another localized scenario is that of a ring-shaped traction profile of a certain width around the equator, as visualized in Fig.2(b). The motivation to examine this scenario is that in the original work of the surface method, a similar traction profile was exerted on a microsphere when being subjected to phagocytosis.[29] We choose a Gaussian traction distribution

with similar parameters to the indenter profile, centered around the equator at $\pi/2$:

$$T_r(\theta) = T_0 \cdot \begin{cases} -\exp(-(\theta-\pi/2)^2/(2\theta_0^2))\sin(\theta) & \text{for } 0 \leq |\theta-\pi/2| \leq \theta_{\text{cut}} \\ 0 & \text{for } |\theta-\pi/2| \geq \theta_{\text{cut}} \end{cases} \tag{55}$$

$$T_\theta(\theta) = T_0 \cdot \begin{cases} -\exp(-(\theta-\pi/2)^2/(2\theta_0^2))\cos(\theta) & \text{for } 0 \leq |\theta-\pi/2| \leq \theta_{\text{cut}} \\ 0 & \text{for } |\theta-\pi/2| \geq \theta_{\text{cut}} \end{cases} \tag{56}$$
